# A LoRaWAN-Enabled Capacitive Sensor Network for Soil Moisture and Groundwater Monitoring

**DOI:** 10.3390/s26206558

**Published:** 2026-10-16

**Authors:** Saman Abdanan Mehdizadeh, Jamal Mohammadi Moalezadeh, Yiannis Ampatzidis

**Affiliations:** 1 Department of Mechanics of Biosystems Engineering, Faculty of Agricultural and Rural Development, Agricultural Sciences and Natural Resources University of Khuzestan, Ahvaz 63417-73637, Khuzestan, Iran; 2 Sugarcane & By-Products Development Research and Training Institute of Khuzestan, Ahvaz 61348-11169, Khuzestan, Iran; 3 Agricultural and Biological Engineering Department, Southwest Florida Research and Education Center, Institute of Food and Agricultural Sciences (IFAS), University of Florida, 2685 SR 29 North, Immokalee, FL 34142, USA; i.ampatzidis@ufl.edu

**Keywords:** IoT, LoRaWAN, precision agriculture, precision irrigation, solar energy harvesting

## Abstract

Water scarcity requires low-cost systems capable of continuously monitoring soil water and groundwater dynamics in agricultural fields. We developed a LoRaWAN-enabled sensing platform integrating a wire-based interdigital capacitive soil-moisture probe fabricated on a PMMA substrate and a U-shaped PTFE-insulated capacitive groundwater probe installed in a perforated PVC tube. Both sensors are interfaced with an ESP32 and communicate through 868 MHz LoRaWAN, with cloud-side calibration for soil moisture. Soil-moisture calibration against the gravimetric reference at 15, 30, and 45 cm yielded R^2^ values of 0.975–0.988 and RMSE values of 1.08–1.66% *v*/*v*. Freshwater groundwater calibration over the measured 0–200 cm range yielded R^2^ = 0.99946 and RMSE = 1.35 cm. The salinity experiment comprised 990 raw observations from 12 sensor probes and 12 solution batches. Outcome-independent robust QC flagged 60 observations (6.06%), leaving 930 observations for analysis. The pooled quadratic calibration achieved R^2^ = 0.99771 and RMSE = 3.02 cm, while EC-specific models yielded R^2^ values of 0.99852–0.99892 and RMSE values of 2.08–2.45 cm. Repeated-measures analysis showed significant main effects of depth and EC but no statistically significant Depth × EC interaction. The maximum mean relative ADC deviation from the EC = 0.5 dS m^−1^ reference was 1.82% at EC = 4.0 dS m^−1^ and 200 cm. Field validation quantified linear association, bias, and agreement with reference instruments. Communication exceeded 90% packet delivery at 8 km in open terrain and 5 km in dense sugarcane under the tested conditions. Routine scheduled sensing required 16.60 mWh day^−1^ with BLE disabled.

## 1. Introduction

Sustainable agricultural production in the face of escalating water scarcity demands precise, real-time monitoring of soil water dynamics. Soil moisture content (SMC) is a critical variable governing irrigation scheduling, crop water uptake, nutrient transport, and ultimately, yield optimization [1]. While traditional gravimetric sampling remains the reference standard for SMC determination, its destructive nature, labor intensity, and inability to provide continuous data render it impractical for operational farm management [2]. Electromagnetic techniques such as time-domain reflectometry (TDR) and frequency-domain reflectometry (FDR) provide non-destructive, in situ measurements of soil water status and are available in both research-grade and lower-cost implementations. Recent studies have demonstrated that compact and commercially available low-cost instruments can be used for TDR/FDR-related measurements of soil water content and electrical conductivity. Consequently, the practical suitability of an electromagnetic sensor depends not only on purchase cost but also on calibration requirements, power consumption, signal processing, installation complexity, durability, and deployment scale [3,4]. In this context, low-cost capacitive architectures remain attractive when the sensing hardware can be fabricated and calibrated for the intended soil and field conditions.

Capacitance-based soil moisture sensors have emerged as a compelling alternative, leveraging the pronounced dielectric contrast between water (relative permittivity ε_r_ ≈ 80) and dry soil matrices (ε_r_ ≈ 2–6) to infer volumetric water content from measurable changes in capacitance [5]. These sensors benefit from simple construction, low power consumption, and compatibility with wireless sensor networks (WSNs), making them well-suited for distributed agricultural monitoring [6,7]. Recent innovations in printed circuit board (PCB) fabrication have enabled cost-effective interdigital fringe-field electrode designs that enhance sensitivity while maintaining robustness [8]. Recent studies have demonstrated that PCB-based capacitive sensors calibrated against gravimetric references can achieve coefficients of determination (R^2^) exceeding 0.80 and relative errors below 6% across multiple soil depths, making them viable for field-scale irrigation management [4]. However, most reported capacitive sensors rely on standard PCB substrates (e.g., FR4), which may not be optimal for field deployment in corrosive, abrasive soil environments. Specifically, the epoxy-glass weave structure of FR4 is inherently hygroscopic; prolonged exposure to soil moisture causes water ingress that alters the substrate’s baseline dielectric permittivity and induces severe measurement drift [5]. Furthermore, micro-cracks in the FR4 solder mask can expose copper traces to the electrolytic soil solution, accelerating galvanic corrosion and eventual sensor failure [9]. FR4-based capacitive probes are widely used because of their low manufacturing cost and convenient fabrication. However, the long-term field behavior of a particular FR4 sensor depends on the substrate, metallization, protective coating, sealing, electrode geometry, and environmental exposure. Moisture ingress and environmental degradation can affect baseline stability and long-term performance in some constructions. Accordingly, the selection of PMMA and PTFE in the present design was motivated by the intended mechanical and environmental operating conditions, while their performance relative to alternative substrate and electrode constructions requires controlled comparative testing. Finally, many IoT-based irrigation systems employ short-range communication protocols (Wi-Fi, Bluetooth, Zigbee) that cannot span the large, often topographically complex areas characteristic of agricultural holdings [10].

Another critical gap in current monitoring systems is the lack of integrated subsurface water profiling. Although recent advancements have enabled multi-depth soil profiling [6,7], these commercial and academic systems typically terminate at the root zone and do not integrate groundwater table monitoring. Consequently, understanding aquifer recharge and drainage requires separate, expensive piezometer installations (e.g., pressure transducers or acoustic loggers) that operate on different communication protocols. Taken together, the literature establishes that low-cost capacitive soil-moisture sensing, multi-depth soil profiling, groundwater-level monitoring, and long-range IoT communication are each technically feasible. However, these functions are commonly implemented as separate sensing and monitoring subsystems. Soil-moisture systems generally focus on the root zone, whereas groundwater observations are typically obtained using dedicated piezometers or pressure-based and acoustic instruments. This separation increases installation and maintenance requirements and makes it more difficult to interpret root-zone water availability together with groundwater dynamics within a common monitoring framework. A unified system capable of simultaneously measuring soil moisture at multiple depths and groundwater table position would therefore provide farmers and hydrologists with a more complete picture of the soil–water continuum at a fraction of the cost and complexity, while maintaining low power consumption, long-range communication, and field-deployable calibration.

To address this gap, the present study develops and validates a unified soil–groundwater monitoring platform. The principal contribution is not the individual use of established technologies, such as the ESP32 microcontroller [2], LoRaWAN architecture [4], or basic capacitive sensing principles using TLC555 oscillators [11,12], but their coordinated implementation in a sensing architecture specifically designed to characterize the subsurface water continuum. The platform combines (i) a custom wire-based interdigital capacitive soil-moisture probe fabricated on a PMMA substrate for multi-depth root-zone measurements, (ii) a U-shaped coaxial capacitive groundwater-level sensor in which the water column forms part of the sensing electrode configuration, and (iii) a LoRaWAN-enabled cloud architecture that separates sensor data acquisition from calibration and processing functions. This architecture enables soil-moisture and groundwater measurements to be acquired within the same wireless monitoring framework rather than through independent sensing systems.

The contribution further extends beyond functional system integration through quantitative metrological characterization. For the soil-moisture sensor, depth-specific calibration relationships are established and statistically evaluated to determine whether calibration behavior varies among the investigated depths. For the groundwater sensor, the effects of electrical conductivity are explicitly investigated by comparing pooled and EC-specific calibration models, evaluating sensor-level generalizability, and quantifying prediction error across the tested depth and conductivity ranges. Groundwater measurements are additionally compared with an independent dip-meter reference using regression, paired statistical analysis, and Bland–Altman agreement analysis. At the system level, LoRaWAN communication performance is characterized under different spreading factors and field conditions, while energy consumption is quantified under the actual duty-cycled operating mode. These experiments provide a combined assessment of sensing accuracy, calibration stability, sensor variability, communication reliability, and energy demand rather than demonstrating connectivity alone.

The specific objectives of the study are therefore to: (1) design, fabricate, and calibrate custom capacitive sensors for multi-depth soil-moisture and groundwater-level measurement; (2) quantify depth-related effects on soil-moisture calibration and conductivity-related effects on groundwater-level measurement; (3) develop the embedded, wireless, and cloud architecture required for integrated field monitoring and remotely managed calibration; and (4) validate the complete platform through laboratory and field experiments covering measurement error, calibration stability, sensor-to-sensor consistency, independent reference agreement, communication performance, energy consumption, and long-term field operation. The resulting contribution is a unified and quantitatively characterized soil–groundwater monitoring platform intended to reduce the separation between root-zone and groundwater observations in agricultural water management.

## 2. Materials and Methods

### 2.1. System Architecture and Network Topology

The monitoring system employs a LoRaWAN star-of-stars topology to balance coverage, energy efficiency, and data integrity across large agricultural fields. The architecture comprises three principal layers: (i) battery-powered end-devices (child nodes) deployed at measurement points; (ii) a single LoRaWAN gateway (UG67, Ursalink/Milesight, Xiamen, Fujian, China) serving as the transparent radio-to-IP bridge; and (iii) a cloud-based backend for data aggregation, processing, and visualization.

**Figure 1:**
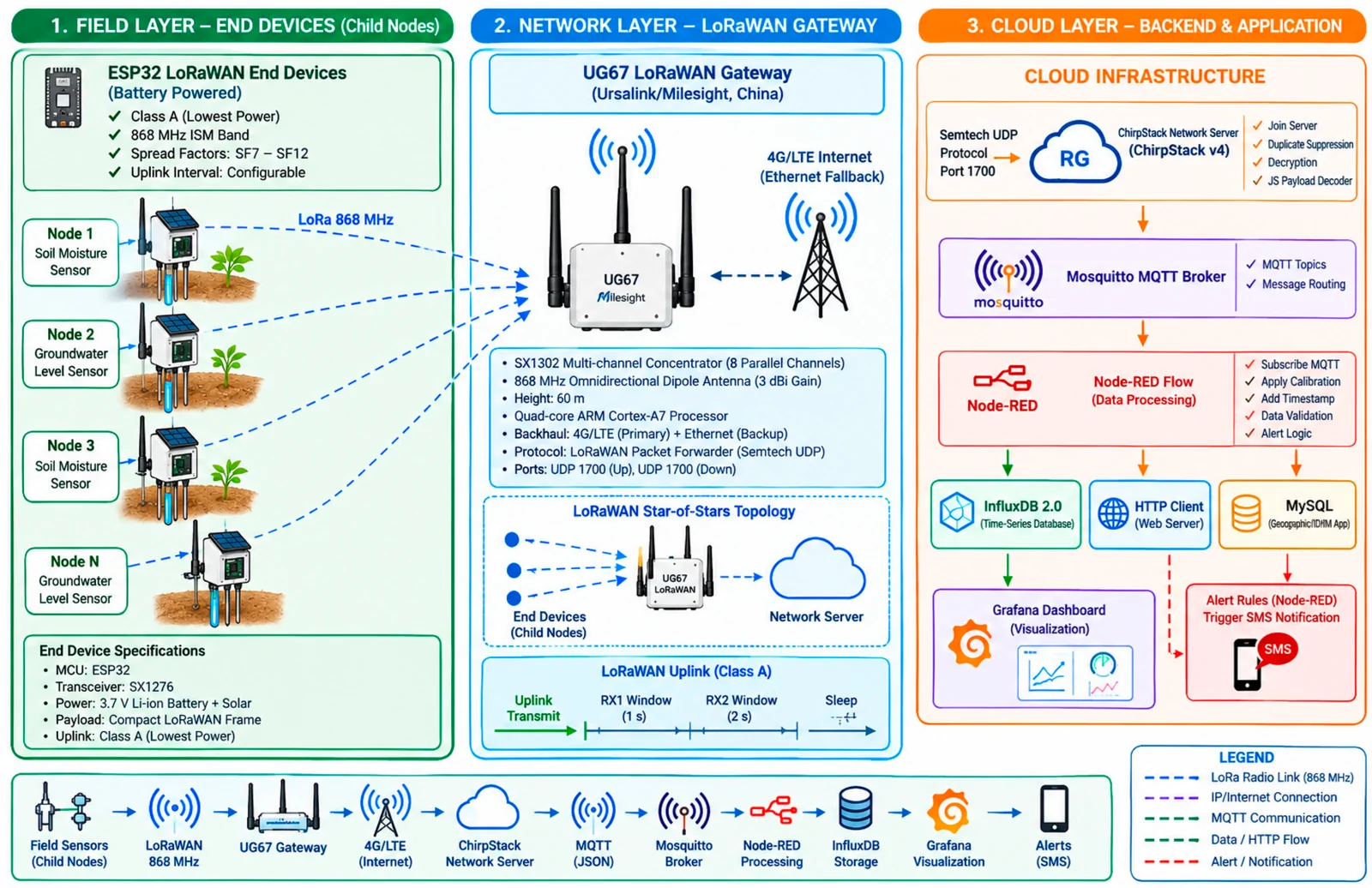
Schematic diagram of the IoT-based soil moisture and groundwater level measurement system.

Each child node is an ESP32-based platform that acquires sensor data, formats it into a compact LoRaWAN payload, and transmits uplinks over the 868 MHz ISM band using Class A protocol (lowest power mode). The UG67 gateway, equipped with an SX1302 multi-channel concentrator (Semtech Corporation, Camarillo, CA, USA), an 868 MHz omnidirectional dipole antenna (3 dBi gain, mounted at 60 m height), and a quad-core ARM Cortex-A7 processor based on Arm architecture (Arm Limited, Cambridge, United Kingdom), continuously monitors eight parallel LoRa channels. It connects to the internet via a built-in 4G/LTE module (with Ethernet fallback) and forwards received frames to a private LoRaWAN network server (ChirpStack v4) hosted on a cloud virtual machine. ChirpStack performs duplicate suppression, payload decryption. Application payloads are decoded using a custom JavaScript codec developed for this study and executed within the ChirpStack application-server environment, then publishes structured JSON messages to a Mosquitto MQTT broker. A Node-RED flow subscribes to the MQTT topic, applies sensor-specific calibration equations, timestamps the data, and writes the results to an InfluxDB 2.0 time-series database. Grafana dashboards (Grafana Labs, New York, NY, USA) provide real-time visualization of soil moisture profiles, groundwater levels, battery status, and link quality metrics. Alert rules trigger notifications (SMS) when soil moisture falls below user-defined thresholds (Figure 1).

This architecture ensures that only the gateway requires continuous internet connectivity and mains/solar power, while distributed child nodes operate autonomously on battery and solar power with multi-year autonomy.

### 2.2. End-Device Hardware

Each child node is built around an ESP32-WROOM-32D microcontroller (Espressif Systems, Shanghai, China), selected for its dual-core Xtensa LX6 processor, integrated 12-bit successive approximation ADC (0–4095 digital range), native Wi-Fi and Bluetooth Low Energy (BLE) radios, and multiple ultra-low-power sleep modes (active: ~80 mA; modem-sleep: ~3 mA; deep-sleep: <30 µA). The LoRa transceiver is an SX1276 (Semtech, Camarillo, CA, USA) connected to the ESP32 via SPI, operating in the 868 MHz band with configurable spreading factors (SF7–SF12) and output power (2–20 dBm).

Power is supplied by a 3.7 V, 10,000 mAh Li-Po battery, continuously trickle-charged by a 2 W monocrystalline solar panel through a TP4056 charge management IC with overcharge, over-discharge, and short-circuit protection. A precision voltage divider (100 kΩ:10 kΩ) connected to an ADC channel enables battery voltage monitoring. A TPS22860 load switch, controlled by a GPIO pin, completely disconnects power to the sensor conditioning circuits and LoRa module during deep sleep, minimizing leakage currents.

**Figure 2:**
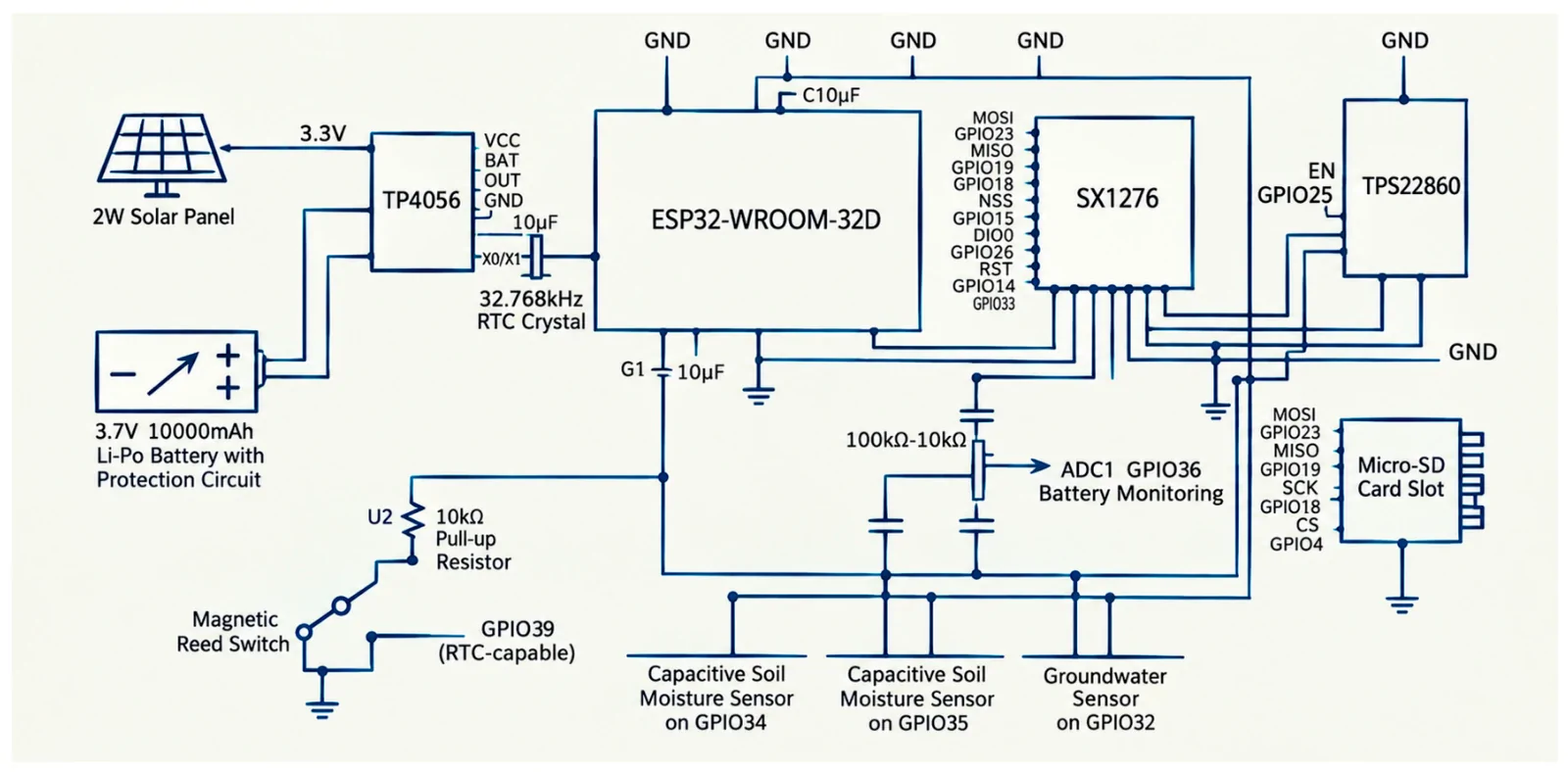
Schematic electrical circuit of child node.

A 32.768 kHz external RTC crystal provides accurate timing for deep-sleep scheduling. A micro-SD card slot (SPI interface) allows local data logging as a fail-safe during communication outages. A magnetic reed switch (Hamlin, normally open) is connected to an RTC-capable GPIO pin configured as a wake-up source; placing a small permanent magnet against the marked enclosure area triggers an immediate hardware interrupt, forcing the ESP32 out of deep sleep for on-demand data acquisition. All electronics are enclosed in an IP67-rated ABS box (155 × 110 × 60 mm) with cable glands for sensor leads and a clearly marked external area for magnetic activation. Figure 2 shows the opened node with PCB, battery, and solar charge circuitry. To substantiate the low-cost claim of the proposed system, a detailed Bill of Materials (BOM) and component-level cost breakdown is provided in Table A1 (Appendix A). The total estimated cost for a single, fully assembled child node—including the microcontroller, LoRa transceiver, custom sensor probes, power management circuitry, solar panel, and IP67 enclosure—is approximately $74.00. The estimated component cost of approximately $74 per fully assembled child node demonstrates the low-cost nature of the proposed platform and places the system below the study’s $150 per-node design target. Direct economic comparison with commercial TDR/FDR systems is not attempted here because purchase prices, configurations, accessories, and deployment requirements vary among products.

### 2.3. Capacitive Soil Moisture Sensor: Wire-Based Interdigital Design on PMMA

**Figure 3:**
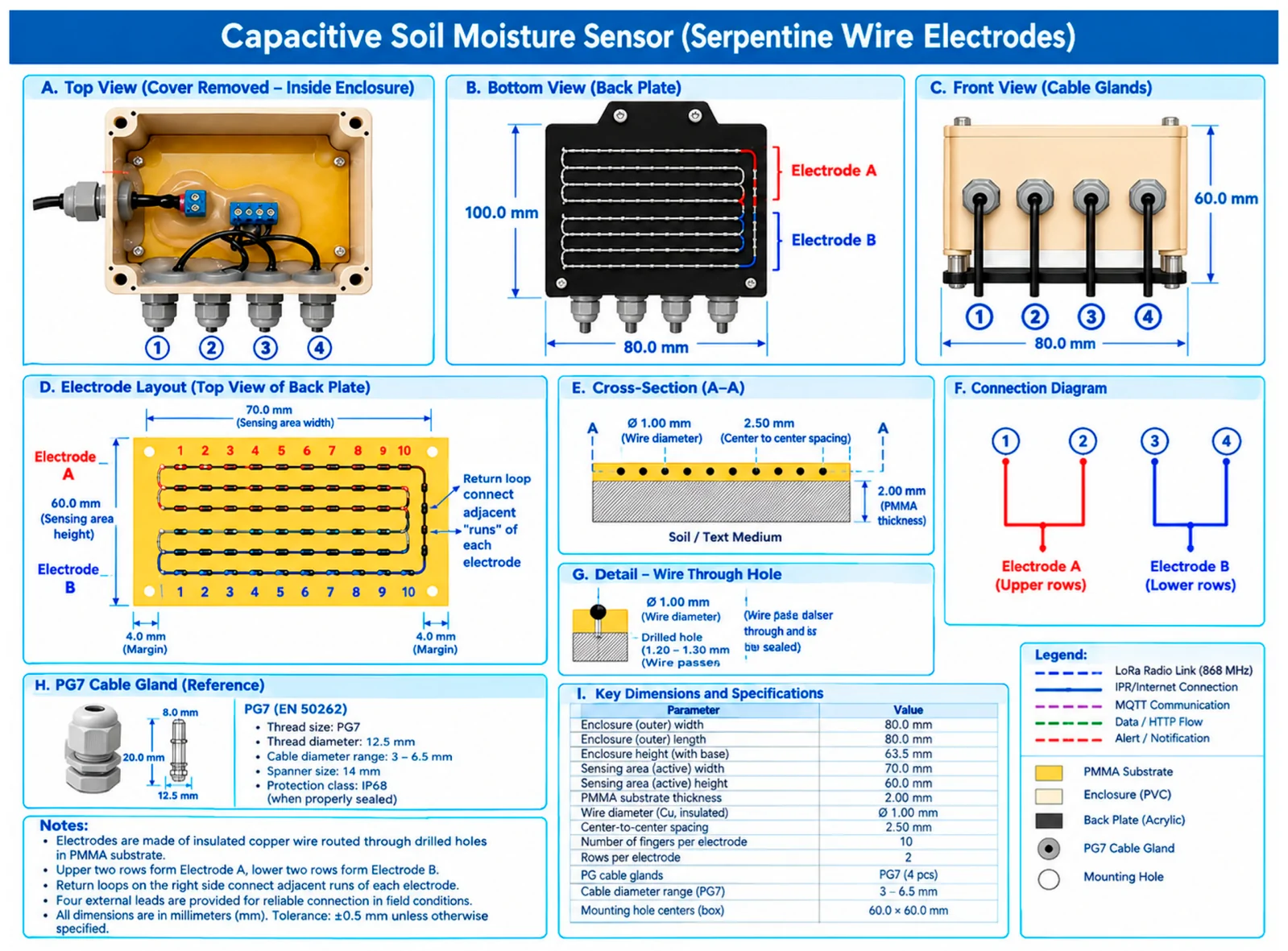
Geometry and construction of the developed capacitive soil moisture sensor.

The soil moisture probe employs a low-cost, insulated-wire interdigital electrode architecture on a transparent PMMA substrate (80 × 20 × 2 mm). Two parallel PTFE-insulated copper wires (conductor Ø 0.25 mm, insulation thickness 0.15 mm, overall Ø 0.55 mm) are assembled by threading them through two rows of laser-drilled holes (hole Ø 0.60 mm, row pitch 1.0 mm, inter-hole spacing 1.0 mm) to form an interdigitated comb of 10 electrode fingers per wire. The first wire is laced in a serpentine pattern through the first row, the second wire through the second row, yielding a center-to-center finger gap of ~1.0 mm and a total sensing length of 50 mm (Figure 3). A self-adhesive copper foil shield (connected to circuit ground) is applied to the reverse side of the PMMA plate to suppress stray capacitance from the connecting loops. Wires are anchored at the rear with epoxy resin and soldered to a two-core shielded cable that carries the signal to the conditioning board.

The PTFE jacket electrically isolates the copper conductor from direct soil contact, thereby reducing the potential for galvanic corrosion and direct DC leakage. The long-term integrity of the insulation and connector interfaces under field conditions remains subject to the duration and environmental conditions of deployment. The sensing capacitance is therefore formed by the series combination of the PTFE insulation layers and the soil’s bulk permittivity. The rigid PMMA substrate provides mechanical support for maintaining electrode separation and presents a smooth, non-porous surface that minimizes soil adhesion. The sensor capacitance C_s_ varies from approximately 5 pF in dry soil to roughly 25 pF in water-saturated soil (values measured at 100 kHz). C_s_ serves as the timing capacitor in an astable oscillator built around a TLC555 timer. Precision resistors R_A_ and R_B_ are chosen so that the oscillation frequency, given by Equation (1) [11]:

(1) $$f=\frac{1.44}{\left(R_{A}+2R_{B}\right)\cdot C_{s}}$$

Frequency lies near 100 kHz for dry soil and falls as moisture increases. To interpret the sensor response at this frequency despite the lossy nature of wet soil, an electrical equivalent circuit model was established. The PTFE insulation acts as a series capacitor (C_PTFE_), while the surrounding soil is represented by a parallel RC network (R_soil_||C_soil_). Accordingly, the sensor should be regarded as a distributed impedance structure operating in a lossy dielectric medium rather than an ideal capacitor. At 100 kHz, the capacitive reactance of the PTFE layer dominates the overall impedance magnitude, substantially reducing the influence of conductive losses associated with soil conductivity. The baseline capacitance values (5–25 pF) were measured using a precision LCR meter (Keysight Technologies, Inc., Santa Rosa, CA, USA) at 100 kHz with a 100 mV excitation signal [12,13]. A LM2917 frequency-to-voltage converter transforms the frequency into a 0–3.3 V signal, which is digitized by a 12-bit ADC in an ESP32 microcontroller. A temperature sensor on the board allows software correction of the water permittivity’s temperature dependence. The entire conditioning circuit is encapsulated in epoxy resin, and the assembled probe has been pressure-tested to a 2 m water column to ensure resistance to prolonged submersion.

### 2.4. Capacitive Groundwater Level Sensor

**Figure 4:**
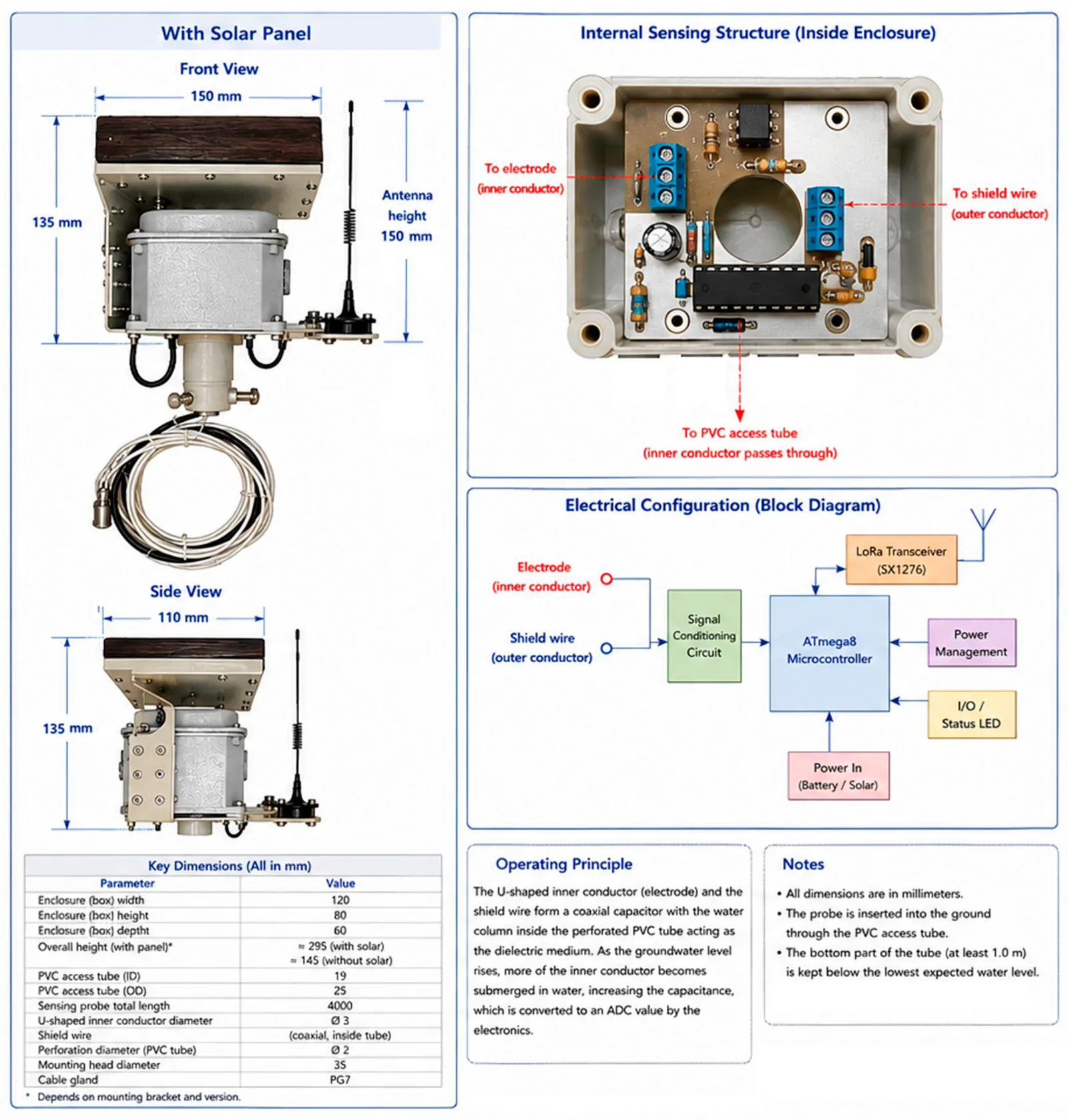
Geometry and construction of the developed capacitive ground water level sensor.

The groundwater level sensor employs a simple coaxial structure built inside a perforated PVC pipe that is buried directly in the soil. The outer protective tube is a standard PVC pipe (inner diameter 19 mm, wall thickness 2 mm, length 2 m) with 6 mm holes drilled at 10 cm intervals to allow groundwater to enter and equilibrate with the surrounding water table. The pipe is installed vertically in a narrow borehole backfilled with coarse sand; it serves simultaneously as the mechanical housing, the water-exchange filter, and the outer boundary of the capacitive structure (Figure 4). It should be noted that the 19 mm internal-diameter PVC pipe serves as the actual sensor housing deployed in the field, and a transparent 19 mm tube of identical internal diameter was utilized during laboratory calibration to maintain geometric consistency while allowing visual monitoring of the water level. The sensor tube was placed inside a larger transparent acrylic tank for controlled filling and observation.

Inside the pipe, two electrodes form the sensing capacitor:

- Inner electrode: a U-shaped PTFE-insulated copper wire (conductor diameter = 1.0 mm, insulation thickness = 0.5 mm, overall diameter = 2.0 mm, total wire length 4 m). The wire descends 2 m, forms a smooth U-turn bend with a minimum radius of 10 mm near the bottom, and returns 2 m to the top, doubling the sensing length. The two ends are connected in parallel at the head of the sensor.
- Outer electrode: a bare stainless steel wire (diameter = 0.5 mm, length = 2 m) that runs down the full depth of the pipe, fixed to the inner wall with small insulating spacers. This wire is electrically bonded to the circuit ground, grounding the entire water column inside the pipe. Because the groundwater is conductive and in direct contact with the bare wire, the water itself becomes a grounded outer conductor that surrounds the insulated inner wire, forming a coaxial structure bounded by the PVC inner radius (R = 9.5 mm).

The capacitor is thus formed by two parallel coaxial segments, each consisting of the insulated inner conductor and the underground water column bounded by the PVC pipe wall. As the water table rises, water (ε_r_ ≈ 80) progressively replaces air (ε_r_ ≈ 1) in the annulus, producing a linear increase in capacitance with the immersed length [14].

For a single vertical leg, the per-unit-length capacitance is well described by the series-dielectric cylindrical capacitor model (Equation (2)) [15]:

(2) $$C_{\mathrm{leg}}=\frac{2\pi \varepsilon_{0}}{\frac{1}{\varepsilon_{\mathrm{PTFE}}}\ln\left(\frac{r_{\mathrm{ins}}}{r_{\mathrm{cond}}}\right)+\frac{1}{\varepsilon_{\mathrm{medium}}}\ln\left(\frac{R}{r_{\mathrm{ins}}}\right)}$$

where *r_cond_* = 0.5 mm, *r_ins_* = 1.0 mm, and *R* = 9.5 mm (the inner radius of the PVC pipe).

The coaxial-cylinder expression used here represents a first-order geometric approximation of the sensing structure rather than an ideal representation of the complete electrical system. In the physical sensor, the PTFE-insulated inner conductor interacts with the conductive water column and the grounded stainless-steel wire, while the PVC boundary, water conductivity, distributed parasitic capacitance, and fringing fields contribute to the overall impedance. Accordingly, the physical sensor is treated as a distributed R–C sensing structure, and its depth response is characterized empirically through calibration.

The total sensor capacitance with two parallel legs is Cs = 2 L immersed. C_leg_, yielding a sensitivity of approximately 1.1 pF per cm of water-level change. The dry-pipe capacitance is about 40 pF, rising to roughly 260 pF when fully immersed over the 2 m length, providing a large dynamic range for the readout electronics. The top-side conditioning board, identical in principle to the soil-moisture circuit, uses the same TLC555 astable oscillator operating at 100 kHz. The conductive water column and the grounded stainless-steel wire form a complex impedance that affects the oscillator response. The PTFE insulation reduces direct conductive loading of the sensing conductor and maintains a predominantly capacitive response; however, it does not eliminate the influence of water conductivity on the overall transfer function. The resulting oscillation frequency is subsequently converted to a proportional voltage by the LM2917 frequency-to-voltage converter.

The electronics are sealed in a waterproof enclosure mounted on top of the PVC pipe. The entire assembly is installed directly in the ground, requiring no separate commercial pressure transducer or acoustic water-level instrument for monitoring the well, which greatly simplifies deployment. The U-shaped insulated wire provides approximately twice the sensing length within a single 2 m pipe, while the bare stainless-steel ground wire serves as the reference electrode without requiring a metallic outer tube. The design retains low material cost, mechanical robustness, easy retrieval for maintenance, and straightforward adaptation to different depths by adjusting the wire and pipe lengths. Temperature compensation and digital filtering were applied to improve measurement stability and repeatability.

### 2.5. End-Device Firmware and Data Flow

At first boot, the ESP32 performs an Over-The-Air Activation (OTAA) join with the ChirpStack network server. Configuration parameters are stored in non-volatile memory and may be updated through a custom BLE maintenance interface. For the routine energy budget reported here, BLE scanning is disabled during scheduled sensing and activated only during explicit maintenance/configuration sessions.

#### Initialization and Configuration

**Figure 5:**
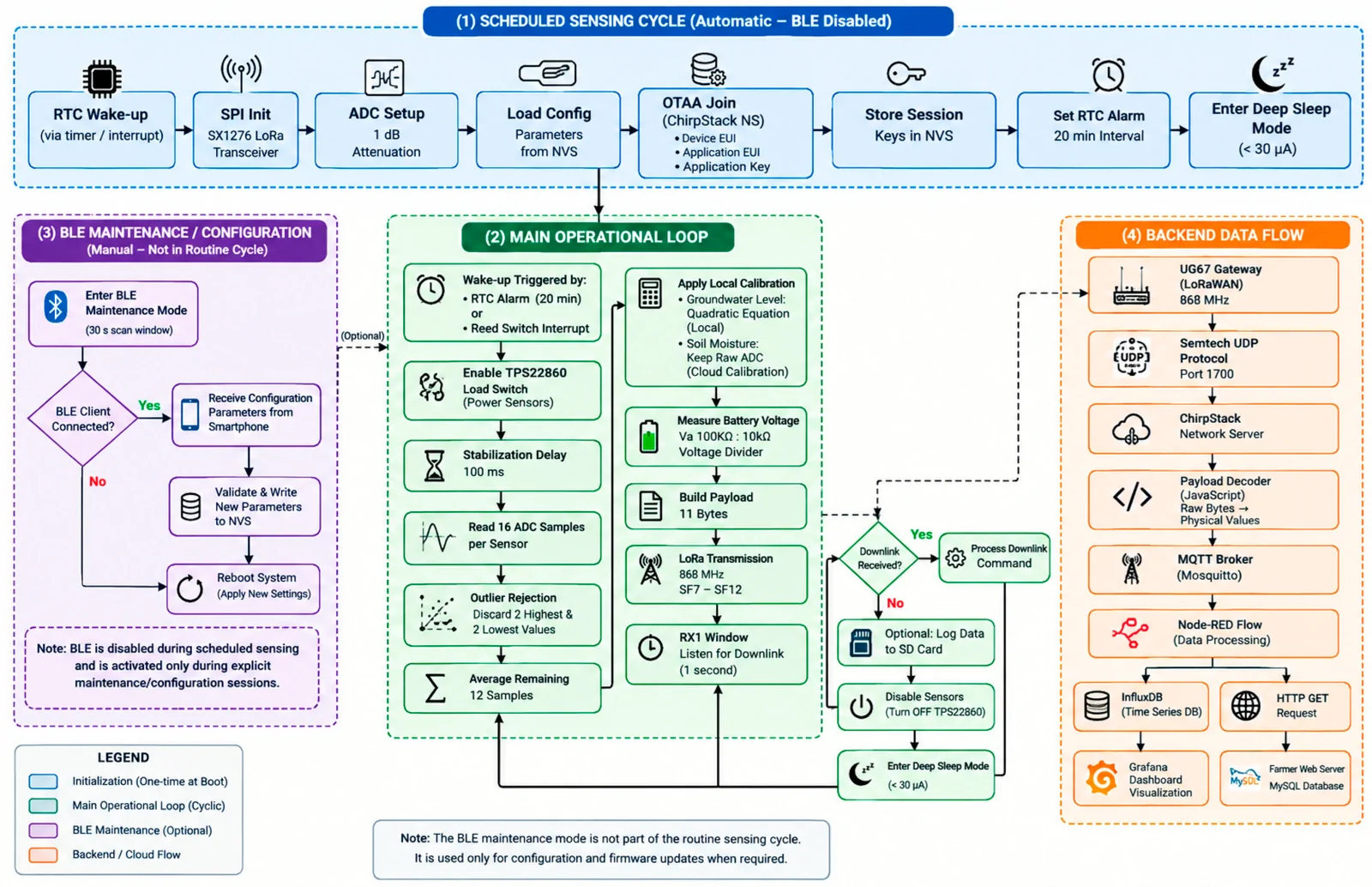
ESP32 LoRaWAN sensor node firmware workflow.

Figure 5 illustrates the complete operational architecture from initialization through data transmission to cloud backend processing. The operation is organized into four functional sections: (1) Initialization sequence (top, blue boxes) showing the boot process including SPI initialization for the SX1276 LoRa transceiver (Semtech Corporation, Camarillo, CA, USA), ADC channel configuration with 11 dB attenuation, reed-switch setup as RTC wake-up source, loading configuration parameters from non-volatile storage (NVS), OTAA join with ChirpStack LoRaWAN network server using the device identifier (DevEUI), application identifier (JoinEUI, formerly AppEUI), and application Key (AppKey), storing session keys in NVS, and setting the RTC alarm for 20 min intervals before entering deep sleep mode (<30 µA power consumption); (2) Main operational loop (center, green box) depicting the cyclical wake-up process triggered by either RTC alarm or magnetic reed switch interrupt, enabling the TPS22860 load switch to power sensors, 100 ms stabilization delay, reading a total of *n* = 16 raw ADC samples per sensor, rejecting transient outliers by discarding the 2 highest and 2 lowest values, averaging the remaining 12 samples to produce a stable measurement, applying the quadratic calibration equation locally for the groundwater level sensor (while soil moisture raw ADC values are retained for cloud-side calibration), battery voltage monitoring via 100 kΩ:10 kΩ voltage divider, building the 11-byte payload, LoRa transmission at 868 MHz with spreading factors SF7-SF12, listening for downlink during the RX1 window (1 s), optional SD card logging, and returning to deep sleep; (3) BLE configuration interface (left side) showing the BLE configuration interface, which is activated only during explicit maintenance/configuration sessions and is disabled during scheduled sensing to minimize energy consumption, allowing smartphone connectivity for wireless parameter updates which are written to NVS followed by system reboot; and (4) Backend data flow (right side, orange boxes) illustrating the complete data pathway from UG67 gateway receiving LoRa frames at 868 MHz, forwarding via Semtech UDP protocol on port 1700 to ChirpStack Network Server, JavaScript payload decoder converting raw bytes to physical quantities, MQTT broker (Mosquitto) message distribution, Node-RED flow processing with dual outputs to InfluxDB time-series database for Grafana dashboard visualization and HTTP GET requests to the farmer’s web server (croplogiot.ir/DIM.php) for MySQL database storage.

**Figure 6:**
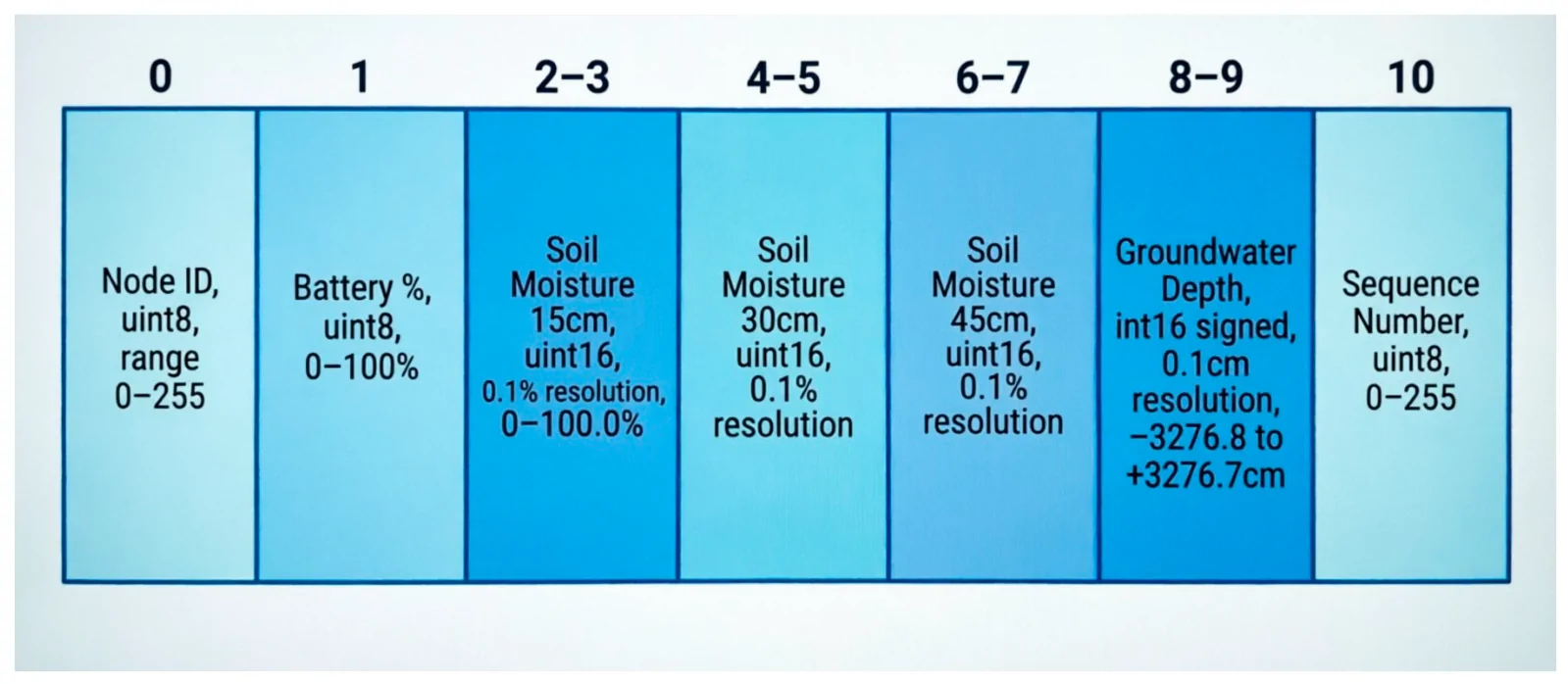
Byte-level layout of the 11-byte application-layer payload.

The application-layer payload comprises an 11-byte packed binary frame, the byte-level layout of which is illustrated in Figure 6. The fields are transmitted in network byte order and include an 8-bit Node ID uniquely identifying the sensor node, an 8-bit battery percentage (0–100%), three unsigned 16-bit soil-moisture ADC readings corresponding to depths of 15, 30, and 45 cm, a signed 16-bit groundwater-depth field encoded with a resolution of 0.1 cm (representable range: −3276.8 to +3276.7 cm; operational measurement range: 0–200 cm), and an 8-bit incrementing sequence number for packet loss detection and ordering (Figure 6). This compact binary encoding minimizes radio airtime and, together with the duty-cycled operation and long-range wireless protocol implemented in the firmware, ensures ultra-low-power operation, reliable long-range communication, wireless configurability, and seamless integration with cloud-based agricultural monitoring platforms.

### 2.6. Sensor Calibration Procedures

#### 2.6.1. Soil Moisture Sensor Calibration (Gravimetric Reference)

The wire-based capacitive probe was calibrated against the standard gravimetric method [16,17]. Bulk soil was collected from the experimental field (loam texture, dry bulk density ρ_b_ = 1.54 g cm^−3^), oven-dried at 105 °C for 24 h, and passed through a 2 mm sieve. Thus, material retained on the 2 mm sieve, corresponding to particles larger than the sieve aperture, was excluded from the calibration samples. Subsamples of 1 kg were prepared in plastic containers. Deionized water was added incrementally to achieve target volumetric water contents (θ^v^) of 5, 10, 15, 20, 25, 30, and 35% (*v*/*v*), with thorough mixing after each addition. Three independent replicate samples were prepared for each combination of packing-depth treatment and target volumetric water content, resulting in 63 paired observations (3 depths × 7 moisture levels × 3 replicates) for the subsequent calibration and ANCOVA analyses. The wetted samples were then left to equilibrate for 2 h at each moisture step. At every target level, the probe was inserted vertically into the center of the soil, and the ADC output was recorded as the mean of readings taken after a 60 s stabilization period. Immediately thereafter, a soil core was extracted adjacent to the probe for gravimetric analysis. The core was weighed wet, oven-dried at 105 °C for 24 h, and reweighed. The gravimetric water content (mass of water per mass of dry soil), expressed as a percentage, was computed using Equation (3) [18]:

(3) $$\theta^{g}=\frac{W_{\omega }-W_{d}}{W_{d}}\times 100\%$$

where W_w_ is wet mass of the soil sample (g), W_d_ is oven-dry mass of the soil sample (g).

The corresponding volumetric water content (volume of water per bulk volume of soil) was obtained from θ^g^ and the known dry bulk density ρ_b_ of soil (Equation (4)) [19]:

(4) $$\theta^{v}=\theta^{g}\times \frac{\rho_{b}}{\rho_{\omega }},\;\rho_{\omega }=1.0\;g\;{\mathrm{cm}}^{-3}$$

To address the inherent heterogeneity of field soils, a rigorous two-step calibration methodology was implemented.

Step 1: Laboratory Baseline Calibration.

The sensor was first calibrated under controlled laboratory conditions using homogenized loam soil collected from the experimental field. Because the soil was homogenized, the three target installation depths (15, 30, and 45 cm) were simulated exclusively by manipulating the bulk density to mimic in situ compaction gradients. A reproducible packing protocol was employed: for each target depth, the exact mass of oven-dried soil required to achieve the target bulk density (ρ_b_) was calculated based on the fixed internal volume (V) of the calibration container (Mass = ρ_b_ × V). The soil was packed in three equal layers, with each layer subjected to a standardized tamping procedure (25 uniform taps with a standardized rubber mallet) to eliminate macropores. The final packed height was verified to ensure the target bulk densities (1.51 g cm^−3^ for 15 cm, 1.54 g cm^3^ for 30 cm, and 1.57 g cm^−3^ for 45 cm) were achieved within a strict ±0.02 g cm^−3^ tolerance. This established the fundamental physical relationship between the raw ADC output and volumetric water content (VWC), proving that compaction gradients alone induce depth-dependent calibration slopes. Consequently, any depth effect observed in this phase specifically reflects these controlled packing-depth treatments and bulk-density manipulations, rather than a direct causal effect of natural soil depth. The specific purpose of this homogenized laboratory calibration is clarified. This initial phase was conducted strictly as a baseline functional validation (proof-of-concept), designed to isolate and verify the sensor’s intrinsic dielectric sensitivity to moisture within a controlled environment. Through the removal of confounding variables inherent to natural soil heterogeneity—such as macropores, root networks, and organic matter—it was definitively demonstrated that moisture changes could be accurately tracked by the sensor’s electronic design. Had this initial validation been performed in undisturbed soil, it would have been impossible for the source of a poor sensor response to be determined, as it could not be established whether the deficiency was caused by a hardware limitation or the complex physical structure of the soil.

Step 2: Field Validation and Texture-Specific Adjustment.

Because the laboratory calibration utilized homogenized soil, it could not account for in situ structural gradients, natural root channels, or localized organic matter variations. Therefore, this laboratory phase serves strictly as a baseline proof-of-concept. The definitive, field-ready calibration is achieved in this step, which is conducted after the sensors are deployed in the field and validated against a commercial TDR350 reference. The cloud-based server then applies soil-specific adjustment factors to the baseline laboratory equation based on the user-selected soil texture. Consequently, a single universal equation is not generated; rather, the system dynamically selects the appropriate depth-specific and soil-type-specific calibration parameters from the cloud database, allowing additional soil-specific calibration profiles to be implemented without firmware modification. Finally, for each depth and soil condition, a linear or non-linear regression model was fitted to the paired dataset of averaged ADC readings and the corresponding gravimetrically determined θ^v^ values, yielding the calibration functions used to convert field sensor readings into volumetric water contents. Model selection followed a hierarchical approach based on three criteria: (i) physical principles governing the sensor operation, (ii) goodness-of-fit metrics (R^2^, RMSE, and residual analysis), and (iii) model parsimony (preferring simpler models unless complexity is justified). For the soil moisture sensor, the relationship between capacitance and volumetric water content is theoretically expected to be approximately linear over the measured range, and linear regression provided excellent fit (R^2^ = 0.975–0.988) with randomly distributed residuals, making more complex models unnecessary. For the groundwater sensor, although the physical capacitance-to-depth relationship is theoretically linear, the frequency-to-voltage converter’s transfer function and fringing field effects at the U-turn bend introduced systematic non-linearity. Quadratic regression was therefore adopted because it: (a) significantly improved R^2^ from 0.992 (linear) to 0.9994 (quadratic), (b) reduced RMSE by 38%, and (c) eliminated the systematic curvature pattern observed in linear model residuals. Model selection was thus guided by both theoretical understanding and empirical validation of residual distributions.

**Figure 7:**
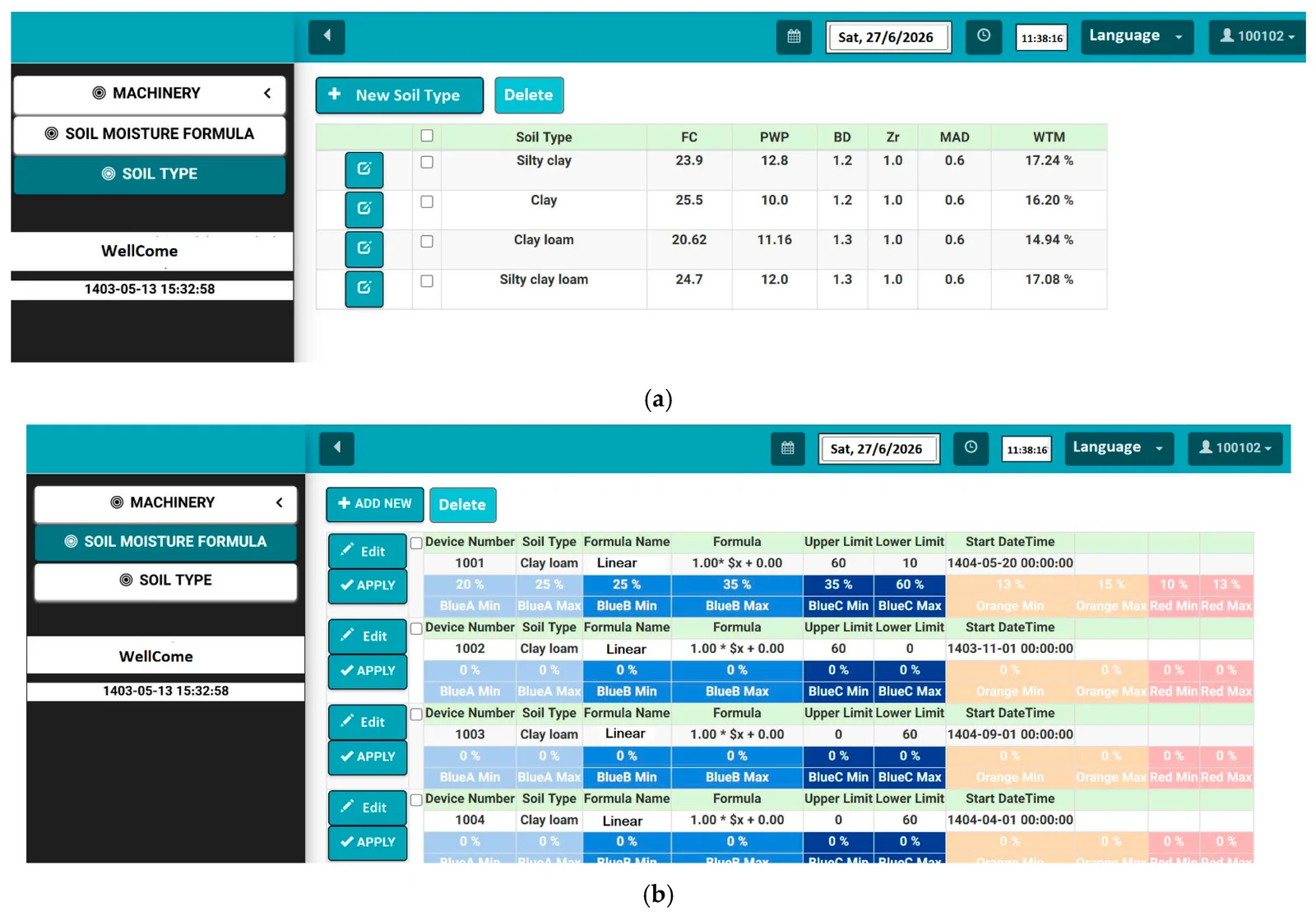
Cloud-based calibration interface. (**a**) Soil texture selection menu for applying predefined calibration profiles. (**b**) Real-time application of depth-specific calibration equations to incoming raw ADC data from sensor nodes.

Rather than embedding soil-moisture calibration coefficients in the constrained microcontroller firmware, the soil calibration parameters are stored and managed on the cloud-based web server. The server interface allows users to select the target soil texture and applies the corresponding depth-specific calibration equation to incoming soil-moisture ADC data. During deployment, each child node transmits the raw 12-bit soil-moisture ADC values for cloud-side calibration, whereas the groundwater depth is converted locally using the quadratic groundwater calibration model before transmission. This architecture separates the soil-moisture calibration logic from the deployed hardware while retaining local groundwater conversion, thereby enabling field recalibration of soil-moisture parameters without requiring firmware modification or physical access to the sensor nodes. Figure 7 shows the web server interface, illustrating the soil-type selection menu (Figure 7a) and the dynamically applied calibration equations (Figure 7b). During deployment, each child node transmits only raw 12-bit ADC values; the server performs real-time conversion to volumetric water content using the user-configured parameters, ensuring operational flexibility and simplifying long-term maintenance across heterogeneous field conditions.

#### 2.6.2. Groundwater Sensor Calibration

Calibration was performed in a transparent acrylic tube (2.1 m height, 10 cm diameter). The U-shaped wire assembly was mounted vertically inside a perforated PVC pipe (19 mm diameter) and placed within the calibration tube. The calibration dataset comprised 41 target water-depth levels from 0 to 200 cm, with three observations at each level, yielding 123 observations for the freshwater calibration. ADC values were rounded to integer 12-bit values before regression. The sensor housing was designed for a nominal operating depth of 200 cm. Three complete fill–drain cycles were performed to assess hysteresis.

Because the sensing circuit contains electronic components with unavoidable component-to-component tolerances, small differences in electrical response were observed among individual sensor assemblies. Accordingly, the calibration procedure was designed to establish a common calibration relationship for the sensor family while also allowing small sensor-specific adjustments during field commissioning. After the general calibration relationship was established, each individual probe could be subjected to a minor field adjustment based on its measured response under known reference conditions. These adjustments were intended to compensate for small electronic and assembly-related variations without changing the underlying calibration model, thereby improving the accuracy and consistency of individual sensors during field operation. This approach allowed the common calibration equation to be retained for the sensor system while accounting for practical sensor-to-sensor variability [20].

For salinity characterization, 30 ADC readings were recorded for each depth–EC condition at EC values of 0.5, 2.0, and 4.0 dS m^−1^ and depths of 0–200 cm in 20 cm increments. The dataset contains 990 observations generated using 12 individually identified sensor probes (S01–S12) and 12 individually identified solution batches (B01–B12). Each sensor was paired with one solution batch at each EC level, and each sensor–batch combination was evaluated across all 11 depth levels. Each sensor–batch–depth–EC series contains 2–3 replicate ADC readings (after QC outlier removal). Prior to inferential testing, replicate readings within each sensor–batch–depth–EC series were averaged, yielding 396 sensor-level condition means. Quality control (QC) was applied before inferential testing and independently of calibration-model performance. For each of the 33 depth × EC conditions, the 30 raw ADC observations were screened using a robust modified Z-score based on the condition median and median absolute deviation (MAD), z∗ = 0.67448975 (ADC − median)/MAD. Observations with ∣z∗∣ > 3.5| were classified as potential anomalous readings.

### 2.7. Lorawan Communication Range, Reliability and Power Autonomy Characterization

To comprehensively evaluate the operational feasibility of the developed wireless sensor network for large-scale agricultural deployment, two interdependent performance dimensions were characterized: (i) long-range communication reliability under realistic field propagation conditions, and (ii) energy consumption and autonomy under duty-cycled operation with solar recharging. These experiments were conducted concurrently to assess the trade-offs between communication range, data reliability, and battery lifetime, critical factors for resource-constrained farming environments.

#### 2.7.1. Communication Range and Reliability Testing

Communication performance was evaluated using a fixed UG67 LoRaWAN gateway (Ursalink/Milesight, China) permanently mounted at an elevation of 60 m on a telecommunications tower, equipped with an SX1302 multi-channel concentrator and an 868 MHz omnidirectional dipole antenna (3 dBi gain). This elevated installation provides enhanced line-of-sight propagation, improved Fresnel zone clearance, and reduced obstruction from vegetation and terrain features—critical advantages for large-scale agricultural coverage [21]. Child nodes, each configured with an ESP32-WROOM-32D microcontroller and SX1276 LoRa transceiver, were sequentially deployed at ground-level positions at distances of 1, 2, 3, 4, 5, 6, 7, 8, 9, and 10 km from the gateway along with predominantly open terrain and minimal vegetation obstruction [22].

At each distance and spreading factor combination (SF7, SF9, SF12), 100 unconfirmed uplinks containing the standardized 11-byte application payload were transmitted at 20 min intervals. PDR, RSSI, and SNR were logged via ChirpStack. Because 100 packets separated by 20 min span approximately 33.0 h from first to last packet, the communication analysis reports packet count and interval separately rather than describing the complete run as a two-hour test window. A link was considered reliable when PDR > 90% [16,17,18,19,20,21,22,23].

#### 2.7.2. Power Consumption and Autonomy Assessment

Current consumption was measured in all operational states using a precision digital multimeter (Keysight 34465A, 6^1/2^-digit resolution) connected in series with the 3.7 V Li-Po battery supply. The following states were characterized:

- Deep sleep: ESP32 in deep-sleep mode with TPS22860 load switch disabling peripherals (<30 µA target).
- Sensor sampling: ADC acquisition, 16-sample averaging with outlier rejection (100 ms duration).
- LoRa transmission: SX1276 active transmission at SF7, SF9, and SF12 with 11-byte payload.
- BLE configuration: 30 s advertisement scan window for smartphone connectivity.
- Solar charging: TP4056 charge management IC efficiency under varying illumination.

Daily energy consumption was computed using Equation (5) [24]:

(5) $$E_{\mathrm{daily}}=\sum_{i}I_{i}\times V_{\mathrm{bat}}\times t_{i}$$

where *I_i_* is current draw in state i, V_bat_ = 3.3 V is the regulated supply voltage, and t_i_ is the cumulative duration of state *I_i_* per day.

Theoretical battery lifetime was estimated as (Equation (6)) [25]:

(6) $$T_{\mathrm{life}}=\frac{C_{\mathrm{bat}}\times V_{\mathrm{nom}}\times \eta_{\mathrm{discharge}}}{E_{\mathrm{daily}}}$$

where T_life_ is the theoretical battery lifetime (days), C_bat_ is the nominal battery capacity (10,000 mAh), V_nom_ is the nominal battery voltage (3.7 V), E_daily_ is the daily energy consumption (mWh), and η_discharge_ = 0.9 accounts for discharge efficiency and voltage cut-off.

A 90-day field autonomy test was conducted with a fully assembled child node deployed in the experimental plot. Battery voltage was logged at each wake-up cycle via the internal ADC and the 100 kΩ:10 kΩ voltage divider. Solar charging efficiency was evaluated by comparing the energy harvested (estimated from panel specifications and local insolation data) against the increase in battery state-of-charge.

### 2.8. Statistical Analysis

All analyses used α = 0.05. Groundwater calibration used OLS quadratic regression on integer 12-bit ADC values. For salinity, quality control was applied within each depth × EC condition using the modified Z-score described above; the complete raw dataset was retained for auditability. After QC, repeated readings within each sensor–batch–depth–EC series were averaged, yielding 396 sensor-level condition means. A two-factor repeated-measures ANOVA was used with water depth and EC as within-sensor factors and sensor identity as the repeated-measures subject. Quadratic groundwater-depth calibration models were fitted to the QC-cleaned pooled dataset and separately within each EC level. For each EC, the pooled and EC-specific models were applied to the same observations; mean prediction bias, MAE, RMSE, and paired Wilcoxon signed-rank tests of absolute errors were calculated. Generalizability was assessed using leave-one-sensor-out cross-validation on sensor × depth cell means. Soil calibration used depth-specific OLS linear regression and ANCOVA with ADC as the covariate, depth-treatment as the fixed factor, and the Depth × ADC interaction. Field validation used paired-sample *t*-tests, mean bias, 95% confidence intervals, Cohen’s d, OLS regression, and Bland–Altman analysis for groundwater. Regression confidence intervals were taken from the fitted OLS models.

For field validation, the paired-sample *t*-test was applied to daily paired reference/capacitive observations. Mean differences, standard deviations of differences, 95% confidence intervals, and Cohen’s d were reported. Groundwater agreement was further evaluated using Bland–Altman limits of agreement, defined as mean bias ±1.96 × SD of the differences.

## 3. Results and Discussion

### 3.1. Groundwater Level Sensor

#### 3.1.1. Salinity and Water-Depth Effects Analysis

The influence of water electrical conductivity (EC) on the capacitive groundwater sensor output was evaluated across three conductivity levels (0.5, 2.0, and 4.0 dS m^−1^) and 11 water-depth levels (0 to 200 cm). The experimental design utilized 12 individually identified sensor probes and 12 independently prepared solution batches. Each sensor was evaluated across all EC conditions and the complete depth sequence. Thirty raw ADC observations were recorded for every depth–EC condition. Because repeated readings from the same sensor–batch–depth–EC series represent correlated electronic measurements rather than independent experimental units, these replicates were averaged within each series prior to inferential testing. An outcome-independent quality-control (QC) screen was applied within each depth × EC condition to identify potential anomalous ADC observations. This procedure identified 60 of 990 raw observations (6.06%) as potential anomalies; the remaining 930 observations were retained for the primary statistical and calibration analyses, while the complete raw dataset was preserved for auditability.

Following QC and replicate averaging, the analysis dataset comprised 396 sensor-level depth × EC cell means (12 sensors × 11 depths × 3 EC levels). Because the same physical sensors contributed observations at every depth and EC level, water depth and EC were modeled as within-sensor factors, with sensor identity serving as the repeated-measures subject. This repeated-measures design effectively controls for fixed between-sensor baseline variations, isolating the specific effects of the experimental treatments. The primary analysis was a two-factor repeated-measures ANOVA of the ADC output, with water depth, EC, and their interaction modeled as within-sensor effects (Table 1).

The repeated-measures ANOVA revealed a very large main effect of water depth on the ADC output (partial η^2^ = 0.9999), indicating that the measured response was dominated by the intended groundwater-depth signal. Electrical conductivity also produced a statistically significant main effect (partial η^2^ = 0.9694), demonstrating that water conductivity contributes measurably to the sensor transfer response. The Depth × EC interaction was not statistically significant, F(20,220) = 0.065, *p* = 1.000, partial η^2^ = 0.0059, indicating that the present data do not provide sufficient evidence that the magnitude of the conductivity effect changes systematically with water depth over the tested 0–200 cm range. Thus, the EC effect is better interpreted as a consistent conductivity-associated shift superimposed on the dominant depth response rather than as evidence of depth-dependent EC sensitivity. This finding also clarifies the physical interpretation of the sensing model: although the coaxial-cylinder expression provides a useful first-order geometric approximation, the actual device behaves as a distributed electrical structure involving the PTFE-insulated conductor, conductive water column, grounded stainless-steel wire, PVC boundary, parasitic capacitance, and fringing fields. The pooled calibration therefore provides a general calibration across the tested conductivity range, whereas EC-specific calibration can reduce prediction error under the tested conductivity conditions. A small depth-dependent residual may nevertheless remain, particularly near 200 cm.

To further characterize the significant EC main effect, EC levels were compared using sensor-level marginal means averaged across the 11 depths. Paired comparisons were conducted to account for the within-sensor pairing, and Holm adjustment was applied across the three pairwise comparisons to control the family-wise error rate. All three pairwise contrasts remained statistically significant after adjustment (Table 2).

The pairwise results confirm that the EC main effect is consistent and highly reproducible across the 12 physical probes. Increasing EC from 0.5 to 2.0 dS m^−1^ shifted the sensor response by approximately 15.6 ADC units, while the shift from 0.5 to 4.0 dS m^−1^ was approximately 36.6 ADC units. Given the sensor’s average sensitivity of approximately 8.5 ADC counts per centimeter, the maximum salinity-induced shift at 4.0 dS m^−1^ corresponds to an apparent depth offset of approximately 4.3 cm before calibration. Over a 200 cm measurement range, this represents a maximum full-scale error of less than 2.2%. Because the Depth × EC interaction was not statistically significant, the present analysis does not provide evidence that the conductivity-associated shift varies systematically with depth over the tested range. The observed conductivity effect can therefore be incorporated through the pooled calibration, while EC-specific calibration provides an option for reducing prediction error when tighter accuracy is required at the tested conductivity levels. However, the descriptive increase in percentage deviation toward 200 cm suggests that a small residual depth-related error may persist at the extreme lower end of the probe. Accordingly, the sensor should be characterized as having good practical tolerance to EC values up to 4.0 dS m^−1^, rather than as completely salinity-independent, ensuring high-accuracy monitoring even in moderately saline agricultural groundwater.

Descriptive statistics for the sensor-level ADC response at each depth and salinity level, computed after outcome-independent QC and averaging repeated readings, are presented in Table 3. For each sensor-batch-depth-EC combination, the repeated readings were averaged to produce a single sensor-level mean; these means were then aggregated to compute the sensor-level descriptive statistics. Table 3 demonstrates the expected monotonic reduction in ADC output with increasing water depth under all three conductivity conditions. At a given depth, the mean ADC response was consistently higher at higher EC, which is consistent with the significant EC main effect in Table 1. At the same time, the sensor-to-sensor dispersion remained comparatively small, as indicated by the exceptionally low CV values below 0.90% across the depth range. Thus, the data support a strong and reproducible depth response with a consistent conductivity-associated offset rather than an unstable or erratic response pattern. The maximum conductivity-associated shift between EC = 0.5 and 4.0 dS m^−1^ remained below 2.0% of the freshwater-reference ADC output at each tested depth, while sensor-to-sensor dispersion remained comparatively small. These results support the use of a pooled calibration for general monitoring over the tested EC range, while EC-specific calibration can be adopted at the lower and upper ends of the tested conductivity range when tighter prediction accuracy is required.

**Figure 8:**
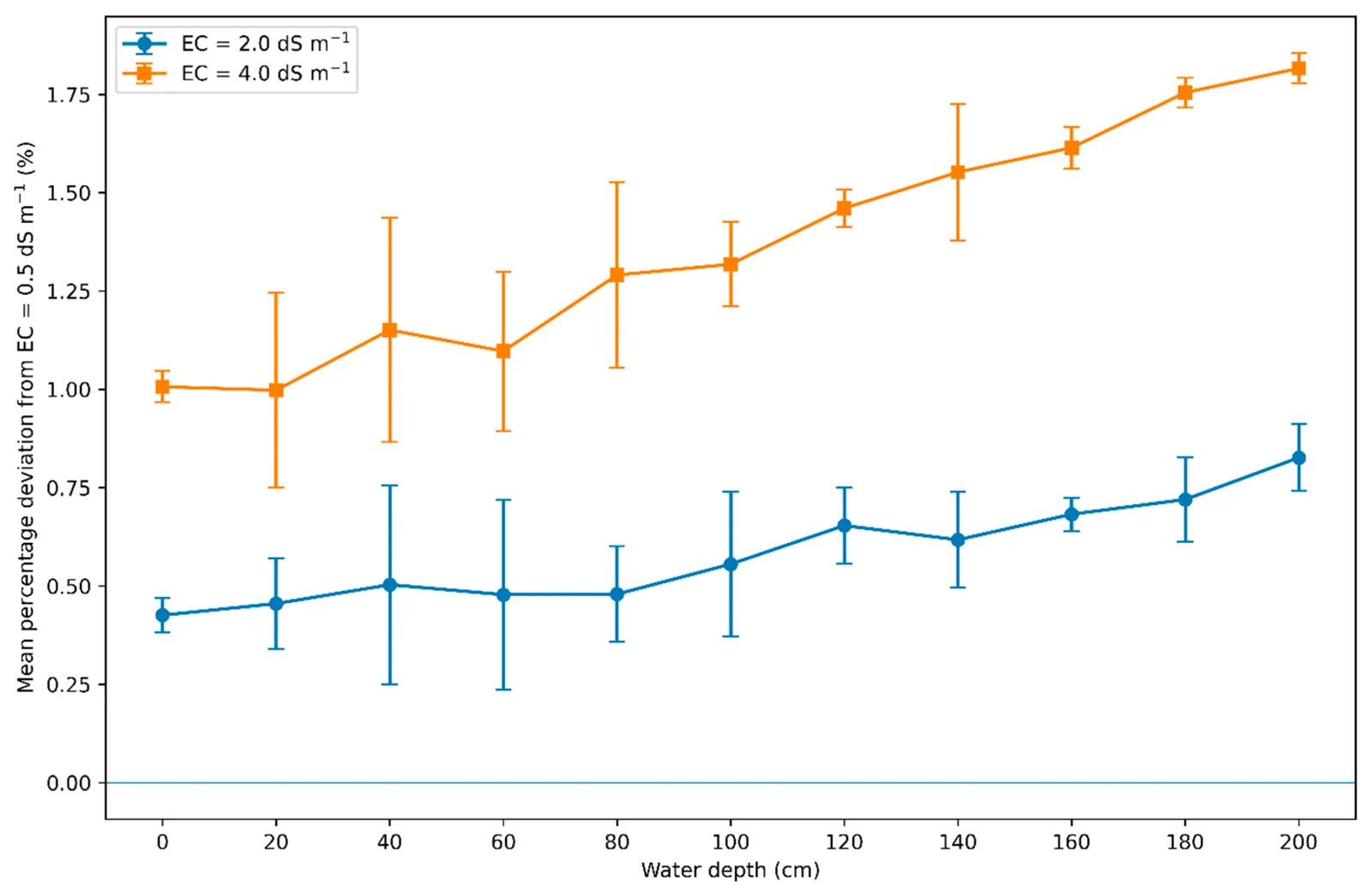
Mean percentage deviation of groundwater-sensor ADC output from the EC = 0.5 dS m^−1^ reference after QC. Points represent the mean across 12 sensor probes after averaging repeated readings within each sensor–batch–depth–EC series; error bars indicate ±SEM.

Figure 8 summarizes the conductivity dependence of the groundwater-sensor response as the percentage deviation in ADC output relative to the EC = 0.5 dS m^−1^ reference. For each sensor–depth–EC combination, repeated ADC measurements were averaged to obtain sensor-level means. Percentage deviations were then calculated individually for each of the 12 sensor probes relative to their corresponding EC = 0.5 dS m^−1^ baseline, thereby controlling for inter-sensor variability in absolute ADC output. Data points represent the mean percentage deviation across sensors (*n* = 12), with error bars indicating ±1 standard error of the mean (SEM). The magnitude of the conductivity-induced shift exhibited a dose-dependent relationship, with deviations at EC = 4.0 dS m^−1^ consistently exceeding those at EC = 2.0 dS m^−1^ across all depths. At EC = 2.0 dS m^−1^, mean percentage deviation increased from 0.43% ± 0.04% (SEM) at 0 cm to 0.83% ± 0.08% at 200 cm depth. At EC = 4.0 dS m^−1^, the corresponding deviations ranged from 1.01% ± 0.04% at 0 cm to 1.82% ± 0.04% at 200 cm, representing an approximately twofold greater shift relative to the lower salinity treatment. The relatively narrow SEM intervals compared to the magnitude of mean deviations indicate high inter-sensor consistency in the conductivity response.

These empirical observations align with the statistically significant main effect of electrical conductivity identified in the within-sensor repeated-measures ANOVA (*p* < 0.001), confirming that elevated salinity systematically increases ADC output. However, the apparent monotonic increase in percentage deviation with depth observed in both EC treatments should be interpreted with caution. The formal Depth × EC interaction term was not statistically significant (*p* > 0.05), indicating insufficient evidence to conclude that conductivity sensitivity varies systematically across the 0–200 cm measurement range. Consequently, Figure 8 serves primarily as a descriptive quantification of the absolute magnitude of salinity-induced sensor bias across the operational envelope, rather than evidence of depth-modulated EC sensitivity. The elevated deviations observed at 200 cm depth, while descriptively notable, do not constitute a statistically validated depth-dependent modification of the sensor’s conductivity response characteristics.

These findings align with and extend recent work in the literature. Ref. [16] reported that PCB-based capacitive soil moisture sensors exhibited relative errors below 6% across a range of field conditions, although their study did not explicitly isolate salinity effects. Ref. [3] evaluated five commercial soil moisture sensors and found that high salinity induced significant noise in TDR315 readings and out-of-range responses in the CS655, highlighting the vulnerability of some dielectric sensing technologies to ionic interference. In contrast, the insulated-wire construction employed in the present design prevents direct electrode-electrolyte contact for the active sensing element, thereby reducing the potential for galvanic effects. Ref. [26] similarly reported deviations below 2.5% for capacitive level sensors at conductivities up to 5.0 dS m^−1^, although differences in sensor construction and experimental conditions should be considered when comparing these results. Ref. [27] documented salinity-induced errors of 1.8 to 3.2% for wire-based capacitive groundwater sensors in agricultural wells; the maximum mean relative ADC deviation observed in the present study was 1.82% at 200 cm and EC = 4.0 dS m^−1^, which falls within the range reported by [27], although the measurement definitions and experimental conditions are not necessarily identical. Collectively, these results indicate that the present sensor exhibits a measurable but limited conductivity-associated shift over the tested EC range of 0.5–4.0 dS m^−1^. A pooled calibration provides a general calibration across this range, while EC-specific calibration can further reduce prediction error under the lower and upper conductivity conditions.

#### 3.1.2. Calibration Curve and Model Fit

The relationship between water depth and sensor output was first characterized under freshwater conditions using quadratic ordinary least-squares (OLS) regression with the integer 12-bit ADC values in the supplied calibration file. The measured calibration dataset spans 0–200 cm and comprises 123 observations collected at 41 target water-depth levels, with three repeated observations at each level. The fitted model is h = c_2_ × ADC^2^ + c_1_ × ADC + c_0_; regression parameters and goodness-of-fit statistics are presented in Table 4.

To quantify the practical effect of electrical conductivity on groundwater-depth estimation, quadratic OLS calibration models were fitted to the QC-cleaned dataset using a pooled model and separate models for each EC level. The pooled model provided a strong overall fit (R^2^ = 0.9977, RMSE = 3.02 cm), while the EC-specific models yielded R^2^ values of 0.9989, 0.9986, and 0.9985, with RMSE values of 2.08, 2.4, and 2.45 cm at EC = 0.5, 2.0, and 4.0 dS m^−1^, respectively (Table 4). Thus, incorporating EC into the calibration improved the fit most clearly at the lowest and highest tested conductivity levels, whereas the improvement at EC = 2.0 dS m^−1^ was comparatively small.

Applying the pooled equation separately to each EC condition revealed a systematic conductivity-dependent prediction bias. At EC = 0.5 dS m^−1^, the pooled model produced RMSE = 3.03 cm and mean bias = +2.15 cm, compared with 2.08 cm for the EC-specific model; the paired Wilcoxon comparison of absolute errors was significant (*p* < 0.001). At EC = 2.0 dS m^−1^, pooled and EC-specific RMSE values were 2.41 and 2.40 cm, respectively, with no significant difference in absolute errors (*p* = 0.170). At EC = 4.0 dS m^−1^, the pooled model produced RMSE = 3.57 cm and mean bias = −2.51 cm, compared with 2.45 cm for the EC-specific model; the paired comparison was significant (*p* < 0.001). A leave-one-sensor-out validation showed mean RMSE reductions of 42.5% at EC = 0.5 dS m^−1^, 0.1% at EC = 2.0 dS m^−1^, and 39.8% at EC = 4.0 dS m^−1^. The corresponding paired Wilcoxon tests of sensor-level MAE gave *p* = 0.00049, *p* = 0.850, and *p* = 0.00049, respectively. Notably, the bias direction reverses across the tested range—positive at low EC, negligible at intermediate EC, negative at high EC—while the magnitude of improvement from EC-specific calibration is symmetric at the two extremes (~40% RMSE reduction) and essentially absent at EC = 2.0 dS m^−1^, the midpoint of the tested range. This pattern, consistent with the interval-RMSE heterogeneity described above, indicates that a pooled calibration is adequate near the center of the conductivity range but should be supplemented with EC-specific or bias-corrected coefficients at the low and high ends if sub-centimeter accuracy is required there. These cross-validated results indicate that conductivity dependence is reproducible across probes and that a pooled calibration is least robust at the lower and upper ends of the tested conductivity range.

**Figure 9:**
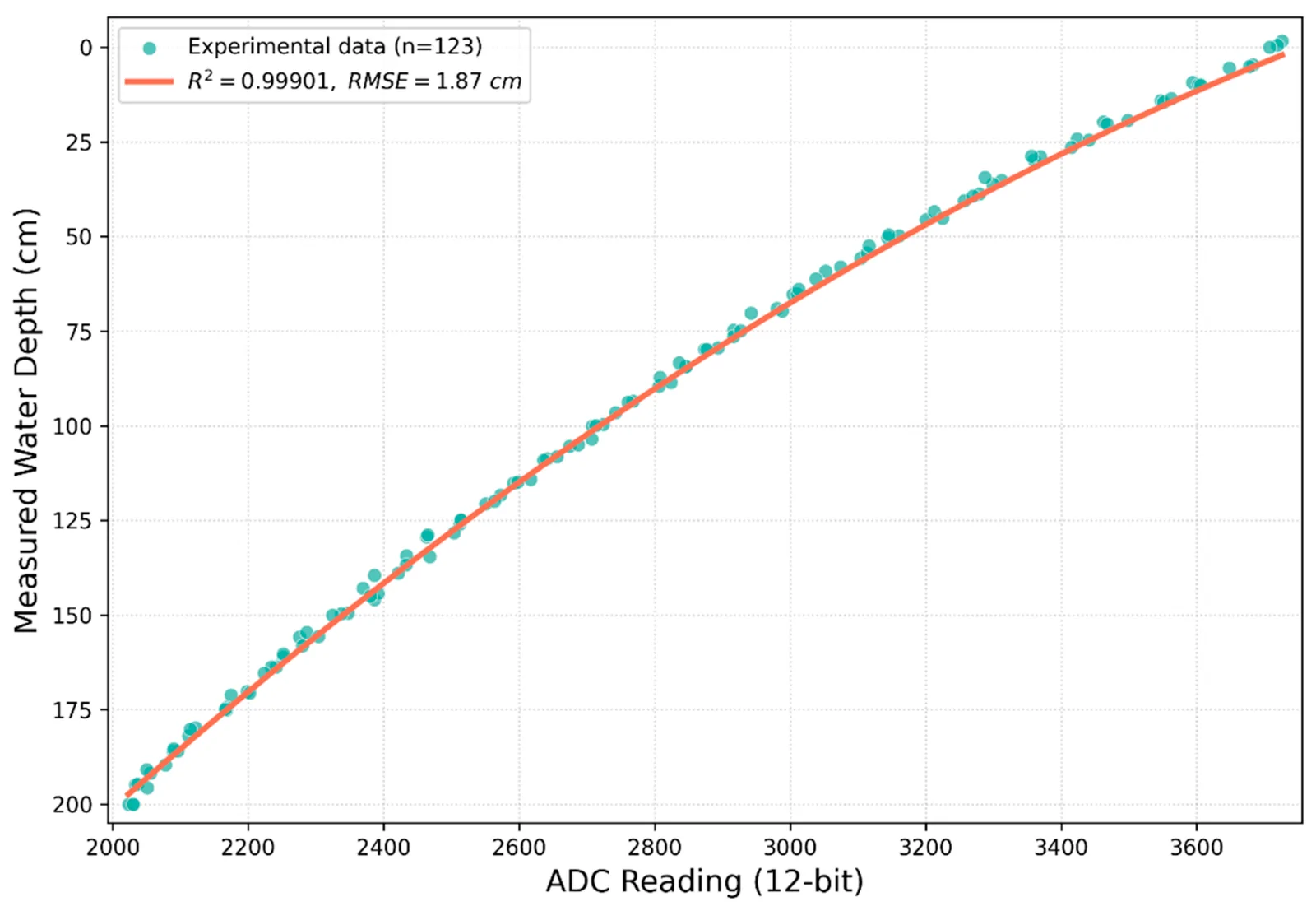
Groundwater calibration observations and quadratic OLS fit using integer ADC values.

Figure 9 presents the measured response of one representative sensor selected from the 12 sensor probes used in the groundwater experiments. The dataset comprises 123 observations collected at 41 water-depth levels from 0 to 200 cm, with three repeated readings at each depth. The sensor exhibited a strong and monotonic decrease in ADC output with increasing water depth, indicating a stable inverse relationship between sensor output and water level. The general quadratic calibration equation provided an excellent agreement with the observations, with R^2^ = 0.99901, RMSE = 1.87 cm, and MAE = 1.53 cm. The small scatter around the fitted curve was maintained across the full tested depth range. Because the three readings at each depth were repeated measurements from the same physical sensor, these observations are presented as within-sensor response data rather than as 123 independent experimental replicates. The figure therefore illustrates the consistency of the general calibration relationship for an individual probe, while sensor-to-sensor generalizability is evaluated separately using the multi-sensor analysis.

The combined statistical and descriptive analyses demonstrate that electrical conductivity (EC) produces a measurable but secondary influence on the groundwater sensor output. The significant within-sensor EC effect and the three significant paired contrasts confirm that increasing conductivity causes a reproducible shift in ADC response. In contrast, the nonsignificant Depth × EC interaction indicates that the data do not provide sufficient evidence that the conductivity effect changes systematically with depth. The sensor response is therefore dominated by water depth, while conductivity contributes a comparatively small calibration offset. Because the interaction was not statistically significant, most of this offset can be corrected using a common calibration relationship or a one-point field adjustment. However, the descriptive increase in percentage deviation toward 200 cm suggests that a small residual depth-related error may remain. Accordingly, the sensor should be described as having good practical tolerance to EC values up to 4.0 dS m^−1^, rather than as completely salinity-independent.

The QC procedure further improved the pooled calibration model, increasing R^2^ from 0.9945 to 0.9977 and reducing RMSE from 4.68 to 3.02 cm, corresponding to a 35.4% reduction in predictive error. Because the QC procedure was outcome-independent and the raw observations were retained for auditability, this improvement is consistent with the removal of anomalous measurements rather than selective optimization of the calibration model. Importantly, QC did not change the principal conclusions: water depth remained the dominant determinant of sensor output, EC produced a statistically significant but practically limited shift, and no statistically validated Depth × EC interaction was detected.

While the exact physical mechanisms driving this progressive depth-dependent degradation were not isolated in controlled experiments, several hypotheses can be proposed based on the sensor’s extended transmission line geometry and its interaction with the surrounding dielectric medium. First, while the shielded internal transmission line successfully mitigates ambient electromagnetic interference, the shield itself introduces progressive hardware limitations as it extends to 200 cm. The shielded cable acts as a distributed resistor–capacitor (RC) network, which may accumulate parasitic shunt capacitance between the inner conductor and the grounded shield along its length. This cable-induced impedance could potentially attenuate the high-frequency signal amplitude and alter phase shifts, which might detune the internal oscillator and reduce frequency stability at deeper measurement nodes—a limitation consistent with the reported behavior of cable-connected capacitive and transmission-line-based dielectric sensing systems [28,29].

Second, environmental gradients may exacerbate these hardware limitations. A continuous 200 cm profile traverses a significant thermal gradient. It is hypothesized that because the complex dielectric permittivity of water—specifically its real component, which dominates the capacitive response—is highly temperature-dependent [30], uniform internal temperature-compensation algorithms may fail to accurately correct deep readings where the soil is significantly cooler and thermally more stable than the surface [31]. Furthermore, the TLC555 timer itself exhibits a temperature coefficient of approximately 50 ppm/°C, which, while small, can compound with the dielectric temperature dependence over a 2 m thermal gradient.

Finally, a potential primary physical driver for the increased RMSE in the deepest interval (151–200 cm) may lie in the electromagnetic boundary conditions at the sensor’s U-turn bend. While the straight vertical segments of the insulated wire exhibit a relatively uniform coaxial electric field, it is hypothesized that the bottom U-turn region introduces severe fringing-field distortion. At the bend, the electric field lines likely diverge radially rather than remaining parallel to the pipe wall, effectively increasing the sensing volume and altering the local dielectric weighting function—an effect broadly consistent with reports that access-tube-mounted electromagnetic sensors exhibit non-uniform field penetration and elevated spatial variability [28,29]. This inherent electromagnetic design limitation could cause a non-linear accumulation of error as the water level approaches the bottom of the sensor. Crucially, this physical non-linearity is precisely why a quadratic calibration model was required for the groundwater sensor, rather than a simple linear one. The quadratic equation empirically compensates for the U-turn fringing-field distortion, effectively correcting the mathematical non-linearity and enabling the exceptionally high overall calibration accuracy observed in this study.

Superimposed on these electromagnetic edge effects, minor mechanical stresses induced by increased lateral earth and hydrostatic pressures at a depth of 200 cm could theoretically play a role. While hydrostatic pressure does not measurably thin the rigid PVC housing, it is hypothesized that these continuous compressive stresses could alter the free volume within the polymer matrix, subsequently shifting its inherent dielectric response [32]. More critically, even microscopic mechanical deformations in the PVC tube might compromise the sensor–soil interface, creating slight air gaps or permitting localized water pooling. Because dielectric sensors are exceptionally sensitive to this immediate boundary layer [29], such deformations could further distort the fringing electric field and introduce additional measurement error.

It should be emphasized that the candidate mechanisms outlined above—cable-induced signal attenuation, thermally driven permittivity drift, bend-induced fringing-field distortion, and mechanical stress—were not experimentally isolated in the present study. They are offered as physically plausible explanations for the observed depth-dependent RMSE pattern rather than confirmed causal pathways. Because these mechanisms would be expected to act simultaneously and in the same direction, disentangling their individual contributions would require a dedicated diagnostic study. Such future work could include network-analyzer characterization of the cable-and-oscillator subsystem in isolation from the soil environment, controlled-temperature-chamber trials at fixed water depth to isolate thermal drift, and finite-element electromagnetic modeling of the U-turn geometry, analogous to approaches used to characterize non-uniform field penetration in access-tube probes [28,29]. Such work would also help determine whether the depth-dependent error could be further reduced through design changes—for example, a wider-radius bend, additional shielding near the U-turn, or a two-point field calibration. In the interim, the quadratic calibration model provides an effective, purely empirical solution that captures the net effect of these confounded mechanisms without requiring their individual quantification.

To characterize the practical distribution of calibration error across the operating range, residuals from the QC-cleaned pooled salinity calibration model were aggregated into root-mean-square error (RMSE) metrics across four predefined depth intervals (0–50, 51–100, 101–150, and 151–200 cm). For the depth-stratified analysis, repeated ADC readings within each sensor × depth × EC combination were first averaged to obtain one sensor-level condition mean. Because the 30 raw observations for each depth × EC condition were distributed across the 12 physical sensor probes, individual sensors contributed two or three repeated readings per condition after QC. The RMSE for each depth interval was then calculated from the residuals of these sensor-level condition means against the quadratic calibration model. This depth-stratified analysis showed that RMSE varied across the operational range and salinity levels (Table 5).

The interval results indicate that while pooled-model error is not strictly uniform across the operating range, it does not exhibit a universal, monotonic deterioration with increasing depth. For instance, at EC = 0.5 dS m^−1^, the mean interval RMSE increased modestly from 2.53 cm (0–50 cm) to 2.80 cm (151–200 cm). Similarly, at EC = 4.0 dS m^−1^, the values ranged from 2.81 cm to 3.84 cm. In contrast, the EC = 2.0 dS m^−1^ condition maintained comparatively low error, ranging narrowly from 1.67 to 1.81 cm across all intervals. Consequently, the largest mean interval RMSE occurred specifically under the combined conditions of high conductivity and maximum operating depth, whereas the intermediate conductivity condition exhibited substantially lower error in the same depth range. This pattern is best interpreted as heterogeneity in practical prediction error under specific boundary conditions, rather than evidence of a fundamental, depth-dependent shift in the sensor’s response to water EC.

From a practical standpoint, the overall pooled calibration RMSE of 3.02 cm masks underlying heterogeneity in prediction error across the operating domain. The maximum mean interval RMSE of 3.84 cm, observed in the 151–200 cm range at EC = 4.0 dS m^−1^, identifies a specific boundary condition under which a common calibration is less accurate; conversely, the substantially lower RMSE at EC = 2.0 dS m^−1^ demonstrates that this maximum error cannot be attributed to depth alone. Plausible contributors to this heterogeneity include conductivity-dependent electromagnetic losses, alterations in the effective dielectric boundary conditions of the sensor structure at extreme depths [28,33], and temperature-induced variations in the imaginary component of the dielectric response [30]. However, because temperature and the precise electromagnetic field distribution were not independently quantified in the present experiment, these mechanisms must be treated as plausible hypotheses for future investigation rather than demonstrated causes. Despite this physical heterogeneity, the sensor’s performance remains highly suitable for practical applications. The maximum interval RMSE of 3.84 cm, observed in the 151–200 cm interval at EC = 4.0 dS m^−1^, represents the largest observed prediction error within the tested operating envelope. This magnitude should be considered in relation to the accuracy requirements of the intended groundwater-monitoring application rather than interpreted as evidence of a universal performance threshold, where management decisions and root-zone dynamics typically operate at spatial scales of several centimeters or more [16,29]. Consequently, the sensor can be deployed over its full 0–200 cm operational range without the need for depth-specific error compensation in routine applications. Should higher precision be required in deep installations, a simple depth-based correction factor could be applied, but the present analysis suggests this is unnecessary for the vast majority of agricultural use cases.

### 3.2. Soil Moisture Sensor Calibration and Depth-Dependent Parameter Assessment

The wire-based capacitive soil moisture sensor was calibrated against the gravimetric reference method. Child nodes transmit raw integer ADC values to the cloud backend, where depth-specific calibration equations are applied. Laboratory calibration was conducted at 15, 30, and 45 cm using homogenized, repacked loam soil; because the target depths were represented by different packing conditions, the laboratory analysis evaluates depth-related packing treatments rather than isolating a natural field-depth effect.

#### Depth-Specific Calibration Parameters and Statistical Comparison

Linear regression models of the form θ_v_ = a × ADC + b were fitted to the paired ADC–θ_v_ datasets for each depth. Regression parameters, goodness-of-fit statistics, and 95% confidence intervals are presented in Table 6.

**Figure 10:**
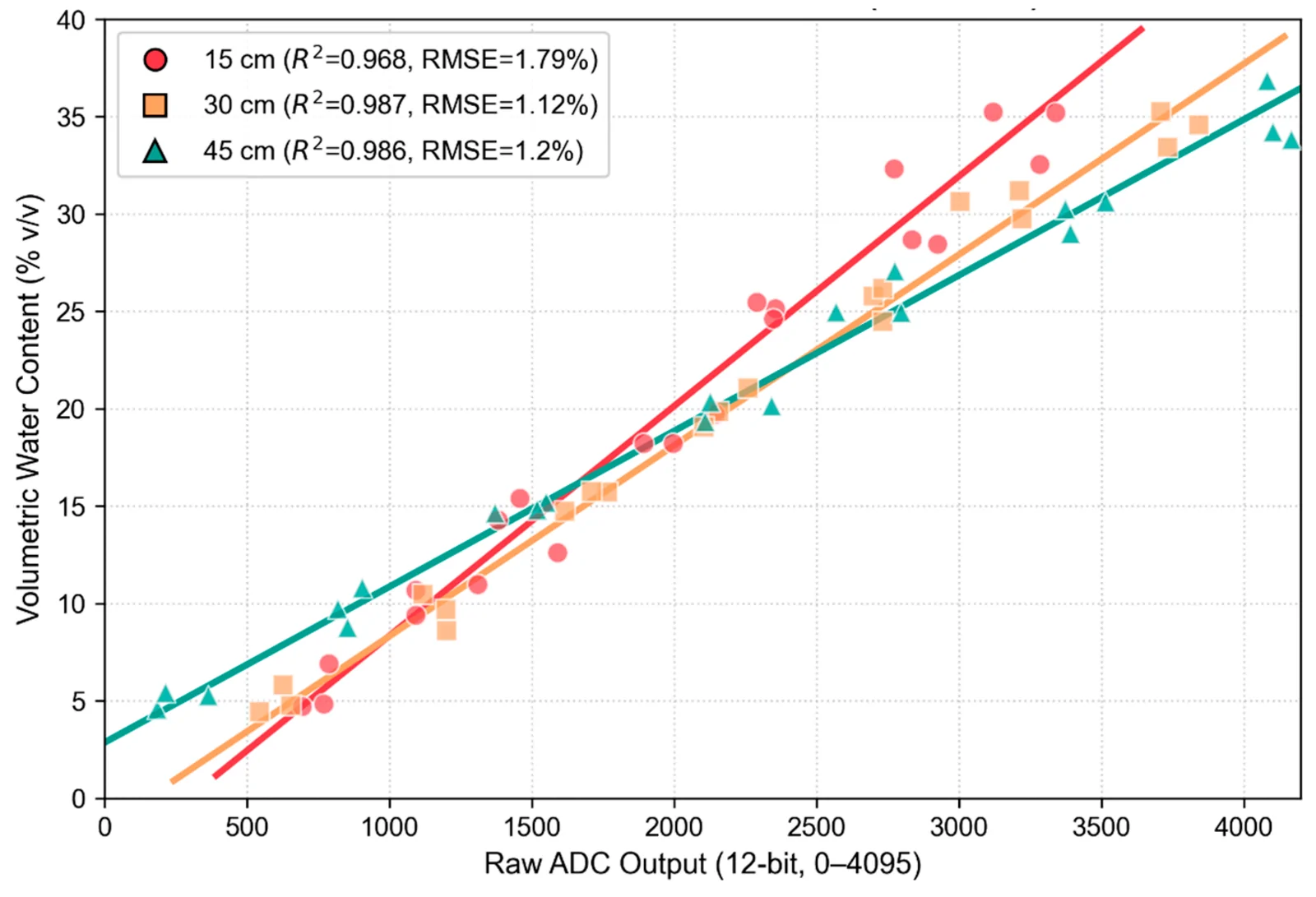
Laboratory calibration scatter plot and fitted linear regression models for the capacitive soil moisture sensor in loam soil at three installation depths (15, 30, and 45 cm).

Figure 10 illustrates the depth-specific calibration models. The slopes decrease from the 15 cm packing treatment to the 45 cm treatment, while all three models retain high R^2^ values. The non-parallel slopes are consistent with the significant Depth × ADC term in the ANCOVA.

To formally test whether calibration parameters differ significantly across depths, a one-way analysis of covariance (ANCOVA) was performed with ADC as the covariate, depth as the fixed factor, and θ_v_ as the dependent variable. Because the Depth × ADC interaction was found to be significant, the full interaction model was retained for the analysis, resulting in 57 residual degrees of freedom (63 total observations minus 6 estimated parameters). The ANCOVA tests two hypotheses: (i) equality of slopes (parallelism), and (ii) equality of intercepts (common origin) after adjusting for the covariate (Table 7).

The fitted model contained 63 observations and six estimated parameters, including the intercept, one ADC covariate term, two depth terms, and two Depth × ADC interaction terms, resulting in 57 residual degrees of freedom. As shown in Table 7, ADC exerted a highly significant effect on θv, F(1,57) = 3281.20, *p* < 0.001, confirming a strong relationship between sensor output and volumetric water content. The depth-treatment effect was also statistically significant, F(2,57) = 8.73, *p* < 0.001. Most importantly, the Depth × ADC interaction was highly significant, F(2,57) = 38.96, *p* < 0.001, demonstrating that the calibration slopes differed among the three tested depth treatments. Thus, the assumption of parallel calibration relationships was not supported by the experimental data.

The fitted laboratory models were: 15 cm, θv = 0.011468·ADC − 3.103; 30 cm, θv = 0.009569·ADC − 1.118; and 45 cm, θv = 0.007889·ADC + 3.108. The corresponding R^2^ values were 0.9749, 0.9881, and 0.9878, with RMSE values of 1.66, 1.08, and 1.09% *v*/*v*. Root density and organic matter were not measured, so their contribution cannot be quantified from this experiment.

The laboratory results support depth-specific calibration within the tested loam packing treatments. Field-scale effects of texture, natural bulk density, organic matter, root channels, temperature, and wetting history remain potential sources of additional variation and require independent field calibration or validation. Field-scale transfer requires additional validation because natural soil profiles differ from the controlled laboratory packing treatments. Soil texture, bulk density, organic matter, root distribution, temperature, hysteresis, and wetting history can alter the dielectric environment surrounding a capacitive sensor and may therefore modify the calibration relationship. The present laboratory results cannot quantify the individual contribution of these factors. Consequently, the depth-specific equations should be regarded as calibration relationships for the tested loam packing treatments, while field deployment should use independently validated parameters for the relevant soil and installation conditions.

The cloud-based architecture provides a practical mechanism for implementing these calibration differences. Incoming raw ADC measurements can be associated with the selected soil texture and deployment depth, and the corresponding calibration parameters can be applied at the server side without modifying the deployed sensor-node firmware. This provides a flexible recalibration mechanism for different deployment conditions. However, this implementation flexibility should not be interpreted as evidence that a single universal calibration equation applies across soil types, depths, and field conditions.

### 3.3. Field Validation Against Reference Instruments

**Figure 11:**
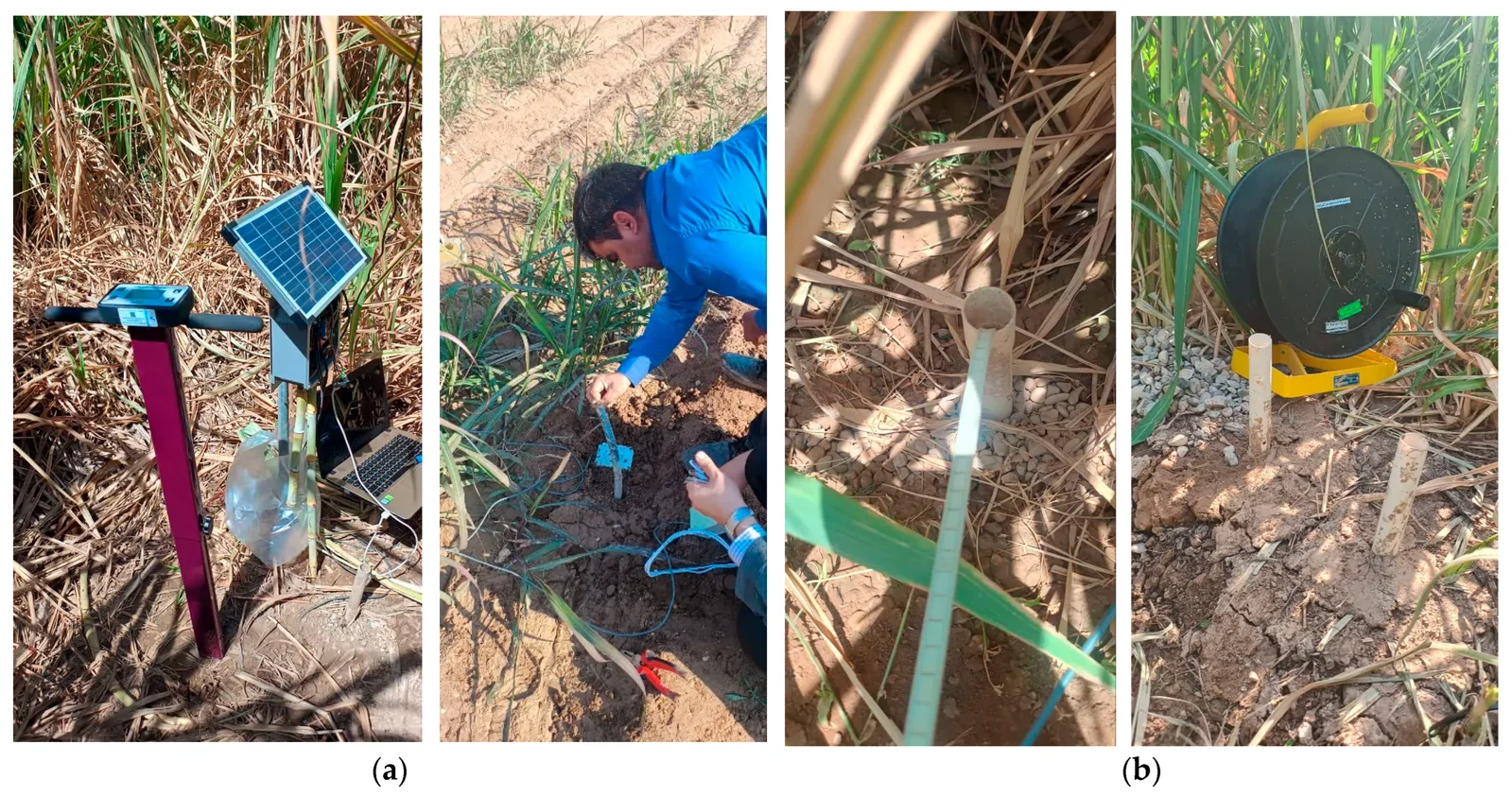
Field deployment and validation setup of the proposed sensing system: (**a**) soil-moisture sensing and field installation; (**b**) groundwater-level sensing and reference-measurement setup.

Field validation compared the capacitive sensors with a TDR350 soil-moisture reference and a manual dip-meter groundwater reference. The supplied soil-validation file contains 90 paired observations at each of 15, 30, and 45 cm, while the groundwater-validation file contains 90 paired observations (Figure 11). Results were evaluated using paired differences, confidence intervals, effect sizes, regression agreement, and Bland–Altman analysis for groundwater.

**Figure 12:**
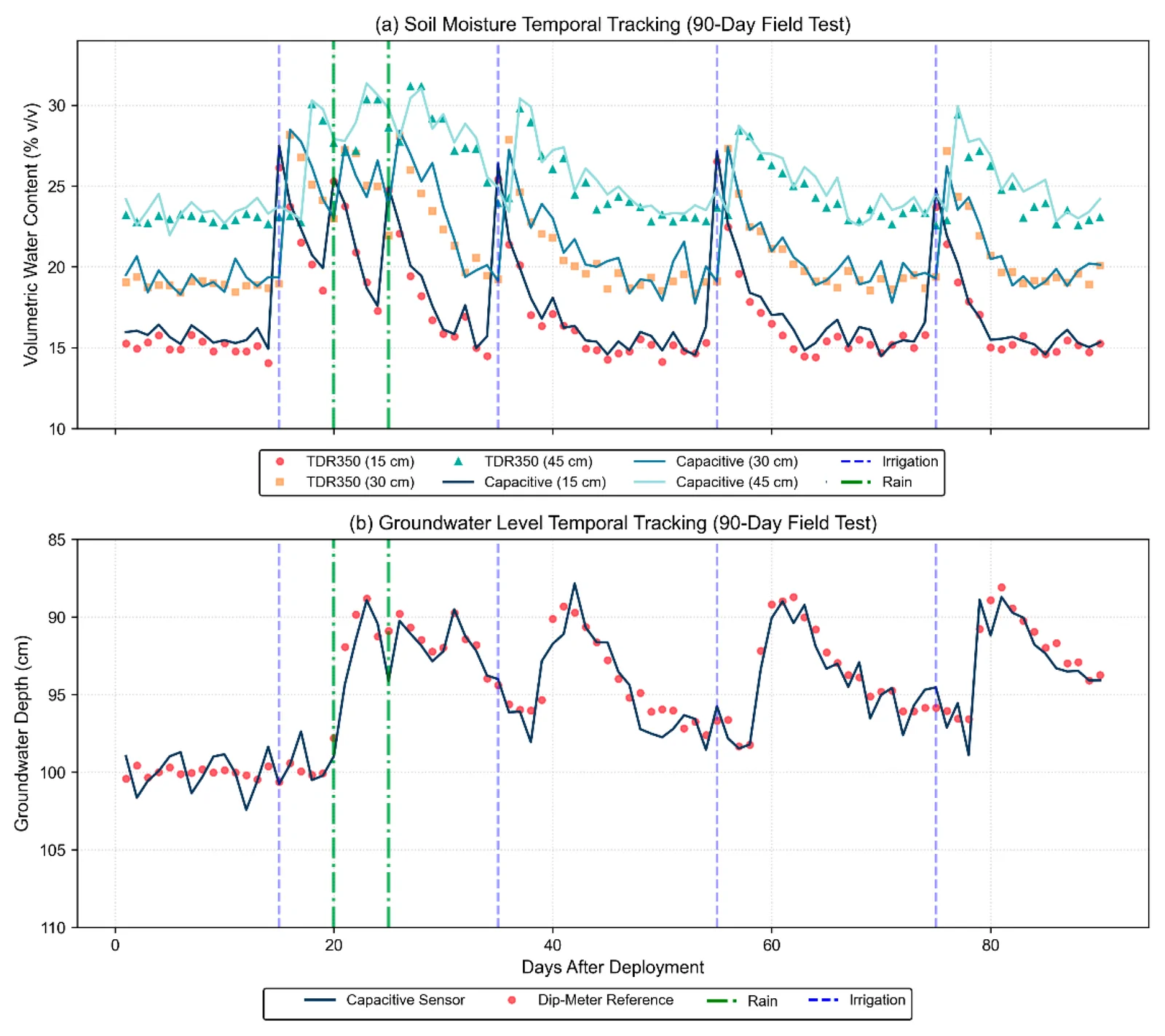
The 90-day field validation time-series comparing the proposed capacitive sensors against reference instruments. (**a**) Soil moisture. (**b**) Groundwater depth.

The temporal evolution of the measurements over the 90-day deployment is presented in Figure 12. The time-series data demonstrate close temporal correspondence between the proposed capacitive sensors and the reference instruments during both scheduled irrigation and unscheduled rainfall events. In the soil moisture profile (Figure 12a), the sensors accurately capture the immediate wetting front at 15 cm, followed by the expected 1- to 2-day delayed and attenuated response at the 30 cm and 45 cm depths, respectively, as the water percolates downward. Similarly, the groundwater sensor (Figure 12b) reliably tracks the gradual recharge of the water table, exhibiting a characteristic 2- to 3-day lag following surface water application. Throughout the 90-day period, no significant sensor drift or temporal offsets were observed, confirming stable performance during the 90-day trial and robust temporal tracking capability of the system under dynamic field conditions.

#### 3.3.1. Soil Moisture Sensor Validation

To evaluate the field performance of the developed sensors, the capacitive soil moisture outputs were compared against concurrent measurements from a commercial TDR350 reference instrument. This comparison utilized paired *t*-tests to assess systematic mean differences and ordinary least-squares regression to quantify the linear association between the capacitive and TDR350 measurements. The resulting descriptive and inferential statistics are summarized in Table 8 and Table 9.

The mean differences between the capacitive sensor and the TDR350 reference were +0.69, +0.31, and +0.47% *v*/*v* at depths of 15, 30, and 45 cm, respectively. While the unadjusted *p*-values were 0.0067, 0.1215, and 0.0363, the application of the Holm-Bonferroni correction yielded adjusted *p*-values of 0.0200, 0.1215, and 0.0726. Consequently, although the difference at the 15 cm depth remained statistically detectable following correction, the overall magnitude of these discrepancies was minimal, as evidenced by the uniformly small effect sizes (Cohen’s d = 0.16–0.29).

Ordinary least-squares regression further demonstrated a strong linear association between the capacitive sensor and the TDR350 reference. The models yielded slopes of 0.961, 0.980, and 0.969 for the 15, 30, and 45 cm depths, respectively. Across the observed volumetric water content range of the TDR350 reference (1.7–46.1% *v*/*v*), the models exhibited coefficients of determination (R^2^) ranging from 0.911 to 0.940, with root mean square errors (RMSE) between 1.88 and 2.33% *v*/*v*.

Although the initial calibration was conducted under controlled laboratory conditions with a 2 h equilibration period at each moisture step, temperature and hysteresis effects remain pertinent when translating these models to field applications. The dielectric properties of both free and bound water are inherently temperature-dependent; for instance, ref. [30] documented significant temperature-induced shifts in soil permittivity at measurement frequencies analogous to those employed by typical capacitive sensors. The 15 cm depth is particularly susceptible to pronounced diurnal thermal fluctuations. Consequently, should dynamic temperature compensation be integrated into the server-side calibration algorithms, the shallowest sensor would likely necessitate the most substantial correction.

Furthermore, moisture hysteresis—the divergence between wetting and drying paths on the soil water retention curve—introduces an additional layer of complexity. While the laboratory calibration adhered to a monotonic wetting sequence, field soils are subject to repeated wetting–drying cycles, which are most pronounced near the surface. The proposed cloud-based architecture, which decouples raw ADC data acquisition from the calibration logic, offers the requisite flexibility to accommodate such depth-specific and temporally variable corrections without necessitating hardware modifications. This approach aligns with established IoT-based sensor recalibration frameworks (e.g., CalibrationTalk) designed to maintain measurement accuracy across heterogeneous deployment conditions.

#### 3.3.2. Groundwater Sensor Validation

Across the 90 paired groundwater validation observations, the mean water depth recorded by the capacitive sensor was 89.14 ± 23.42 cm, compared to 88.74 ± 23.94 cm measured by the reference dip-meter. The mean bias between the two instruments was +0.397 cm, indicating a marginal tendency for the capacitive sensor to report slightly greater depths (Table 10). A paired-samples *t*-test revealed that this difference was not statistically significant at the α = 0.05 level (t(89) = 1.88, *p* = 0.063). The effect size was small (Cohen’s d = 0.20), and the 95% confidence interval for the mean bias (−0.023 to +0.816 cm) encompassed zero, indicating insufficient evidence to conclude a systematic non-zero bias.

Ordinary least-squares regression showed a strong linear association between the capacitive groundwater sensor and the dip-meter reference, yielding h_cap_ = 0.975 × h_dip_ + 2.64, with R^2^ = 0.9934 and RMSE = 1.90 cm (Table 11). The 95% confidence interval for the slope was 0.958–0.992, while the intercept confidence interval was 1.09–4.19 cm. Because the slope confidence interval does not include unity and the intercept confidence interval does not include zero, the regression should be interpreted as evidence of strong linear association with small proportional and additive differences rather than as evidence of exact 1:1 agreement. Bland–Altman analysis gave a mean bias of +0.397 cm and 95% limits of agreement of −3.53 to +4.32 cm, with 93.3% of paired differences within these limits. These results provide complementary estimates of association, bias, and agreement and should not be interpreted as establishing unconditional measurement interchangeability.

Collectively, the field-validation results demonstrate robust agreement and minimal mean bias between the proposed capacitive sensors and their respective reference instruments. Although the paired differences for the groundwater sensor and the 30 cm and 45 cm soil moisture sensors were not statistically significant, a statistically significant bias was detected at the 15 cm soil depth following Holm correction. Nevertheless, the overall effect sizes remained small across all measurement depths, confirming the practical utility of the sensors for field applications while underscoring the necessity of depth-specific calibration protocols.

### 3.4. Energy Efficiency and Operational Lifetime Results

#### 3.4.1. Communication Performance

The communication results show strong short-range performance and increasing dependence on spreading factor with distance and vegetation (Table 12). At 5 km open field, SF9 and SF12 remained above the >90% reliability threshold (96.3% and 95.8%), while SF7 fell to 82.4%. At 8 km open field, SF12 reached 90.3% PDR, whereas SF9 and SF7 fell to 71.6% and 38.1%. In dense sugarcane, SF12 remained above threshold at 5 km (92.1%) but fell to 84.6% at 8 km. The SNR values at 8 km for SF12 were −6.2 dB in open field and −7.8 dB in sugarcane.

For each distance, spreading-factor, and vegetation condition, the communication test consisted of 100 unconfirmed uplinks transmitted at 20 min intervals using the standardized 11-byte application payload. Thus, the nominal interval between consecutive packets was 20 min, and the period from the first to the 100th packet was approximately 33.0 h, calculated as 99 × 20 min. Accordingly, the experiment is not described as a two-hour continuous radio test. PDR, RSSI, and SNR were calculated from the complete set of packets recorded during each scheduled communication run. This distinction ensures that the reported packet count, transmission interval, and test duration are internally consistent.

The measurements demonstrate the expected trade-off between spreading factor, reliability, and airtime. The measured pure on-air time increased from 41 ms at SF7 to 164 ms at SF9 and 655 ms at SF12 for the 11-byte payload (Table 12). The empirical 8 km open-field result should therefore be interpreted as a field performance boundary for the tested 60 m gateway configuration rather than as a universal propagation limit.

The empirical 8 km open-field result should be interpreted as a field performance boundary for the tested 60 m gateway geometry rather than as a universal propagation limit. The detailed Fresnel-zone and two-ray calculations previously stated in the manuscript were removed from the main interpretation because the full set of parameters needed to reproduce them was not documented.

The practical communication guideline is empirical: SF7 is adequate at short range, while SF9 or SF12 should be preferred as distance or vegetation attenuation increases. In dense sugarcane, the tested 8 km point did not meet the >90% criterion even at SF12, so 5 km is the supported reliable range under the tested crop conditions.

#### 3.4.2. Power Consumption and Autonomy

The measured power profile of the ESP32-based child node is presented in Table 13. The node operates in two distinct modes: routine scheduled-sensing mode, in which BLE is disabled, and maintenance/configuration mode, in which BLE is temporarily enabled for smartphone communication. During routine operation, the TPS22860 load switch disconnects non-essential peripherals during deep sleep, resulting in a measured deep-sleep current of 28 ± 3 µA.

For the routine scheduled-sensing cycle, which occurs every 20 min, the measured energy consumption was 829.8 ± 15.2 mJ per cycle, corresponding to 16.60 ± 0.30 mWh day^−1^ for 72 scheduled cycles per day. This value includes deep sleep, sensor acquisition, and LoRa transmission, but does not include BLE activity because BLE is disabled during scheduled sensing. The SF12 LoRa transmission state consumed 705.1 mJ per cycle, with a measured radio-active duration of 1.8 s, including wake-up, PLL locking, and packet handling overhead, compared with a pure on-air time of 655 ms for the 11-byte SF12 packet. The BLE interface is not part of the routine sensing energy budget. When activated for maintenance or configuration, the measured 30 s BLE advertising-scan period consumed 1504.8 mJ at an average current of 15.2 ± 1.1 mA. For completeness, if this 30 s BLE activity were instead executed at every scheduled wake-up, the modeled energy consumption would increase from 829.7 to 2334.5 mJ per 20 min cycle, corresponding to 46.69 mWh day^−1^. Thus, the 16.60 mWh day^−1^ value should be interpreted specifically as the energy requirement of the BLE-disabled routine sensing mode, whereas 46.69 mWh day^−1^ represents the conservative hypothetical case in which BLE scanning occurs at every wake-up.

For a 10,000 mAh Li-Po battery with a nominal energy capacity of approximately 37 Wh and an assumed 90% usable fraction, the idealized autonomy is approximately 5.49 years in BLE-disabled scheduled-sensing mode. Under the hypothetical every-wake-up BLE condition, the same calculation gives approximately 1.95 years. These values are theoretical energy-budget estimates and should not be interpreted as guaranteed field battery lifetimes. A 90-day field autonomy test was conducted using a fully assembled child node in the experimental plot. Battery voltage was logged at each scheduled wake-up, while solar charging was evaluated from the measured system operation and the available photovoltaic input. The energy-budget interpretation therefore distinguishes the routine deployed sensing mode from the intermittent BLE maintenance mode rather than combining both modes into a single headline value.

The deep-sleep current remains an important contributor to the node’s low energy consumption, but total autonomy depends on the operating mode. With BLE disabled during scheduled sensing, the 37 Wh battery at 90% usable capacity corresponds to an idealized autonomy of approximately 5.49 years at 16.60 mWh day^−1^. If the 30 s BLE scan were instead executed at every wake-up, the same energy model would give approximately 1.95 years. The 16.60 mWh day^−1^ value therefore represents the routine scheduled-sensing mode, in which BLE is disabled, whereas the 46.69 mWh day^−1^ value represents the modeled case of BLE activity at every wake-up.

### 3.5. Measurement Device Characteristics, Limitations and Future Work

The developed system combines a PTFE-insulated wire-based soil-moisture sensor, a groundwater-level sensor, an ESP32/LoRaWAN node, and cloud-side soil calibration logic. Soil calibration RMSE ranged from 1.08 to 1.66% *v*/*v*, while the freshwater groundwater calibration achieved R^2^ = 0.99946 and RMSE = 1.35 cm over the validated measurement range. Salinity characterization used 12 individually identified sensor probes and 12 identified solution batches. The raw salinity dataset contained 990 observations; outcome-independent QC flagged 60 observations (6.06%), leaving 930 observations for the primary analysis. The QC-cleaned pooled quadratic calibration achieved R^2^ = 0.99771 and RMSE = 3.02 cm, while EC-specific models achieved RMSE values of 2.08–2.45 cm. Repeated-measures analysis showed significant main effects of depth and EC but no statistically significant Depth × EC interaction. The maximum mean relative ADC deviation from the EC = 0.5 dS m^−1^ reference was 1.82% at EC = 4.0 dS m^−1^ and 200 cm. Field validation demonstrated strong linear association with small mean differences, while groundwater agreement was additionally quantified using Bland–Altman analysis. The groundwater sensor used the same 19 mm internal-diameter sensor housing during laboratory calibration and field deployment; the laboratory sensor tube was placed inside a larger transparent acrylic tank for visual observation. Communication reached threshold-level PDR at 8 km in open field with SF12 and 5 km in dense sugarcane. The 16.6 mWh day^−1^ energy budget applies to routine sensing with BLE disabled.

A further limitation is that the present study did not include a matched sensor-level comparison with a conventional FR4-based capacitive probe or another commercially available wire-based capacitive groundwater sensor. Consequently, the individual contributions of the PMMA substrate, PTFE insulation, and U-shaped groundwater geometry cannot be separated experimentally from the effects of calibration and electronics. Future work should expand the groundwater calibration and validation to additional sensor units, broader environmental conditions, controlled temperature levels, and longer field deployments to further quantify between-unit variability and long-term stability. The communication reliability, although excellent up to 8 km, depends on a single gateway; a hardware or connectivity failure at the gateway severs the entire network, making the system vulnerable in isolated installations unless a redundant gateway or store-and-forward mesh extension is implemented [34,35]. The adaptive data rate (ADR) mechanism, while proven effective, must be tuned carefully to avoid excessive spreading factor oscillations that can waste airtime and energy. Furthermore, the current validation was conducted over a single growing season; long-term sensor drift due to insulation aging, electrode corrosion (especially at connector interfaces), and biofouling of the PVC pipe perforations remains unquantified. Finally, the cost per node, while low compared with commercial TDR/FDR systems, still exceeds the reach of the most marginal smallholder without collective ownership or micro-financing models, and the assembly of the wire-based sensors requires moderate technical skill.

Future work will address these limitations along several axes. A multi-year deployment across diverse soil types and climates will be initiated to quantify sensor drift, assess the durability of the PTFE insulation and PMMA substrate, and establish recalibration intervals. The incorporation of a low-power digital temperature sensor (e.g., DS18B20) at each measurement depth is planned, enabling real-time, depth-specific permittivity correction that is expected to further improve the shallow-sensor accuracy. The salinity experiment will be extended with larger numbers of independently identified sensors and solution batches and a broader EC range to quantify between-unit variability and determine the operating boundary at higher conductivity levels. To improve network resilience, a mesh-capable LoRa protocol (e.g., Meshtastic or a custom flooding algorithm) will be evaluated as a fallback mode that allows child nodes to relay packets when the primary gateway is unreachable. Machine-learning-based recalibration algorithms, similar to the “CalibrationTalk” framework, will be integrated into the cloud backend to periodically adjust calibration parameters using periodic gravimetric spot checks without physical node access. Finally, a streamlined, pre-assembled sensor module and a simplified web-based configuration wizard will be developed to lower the deployment barrier, making the system truly accessible to smallholder cooperatives and extension services.

## 4. Conclusions

The developed system combines a PTFE-insulated wire-based soil moisture sensor, a coaxial groundwater level sensor, an ESP32/LoRaWAN node, and cloud-side calibration logic. Soil calibration RMSE ranged from 1.08 to 1.66% *v*/*v*, and freshwater groundwater calibration over the measured 0–200 cm range achieved R^2^ = 0.99946 and RMSE = 1.35 cm. Salinity characterization used 12 identified sensors and 12 identified solution batches. Outcome-independent QC flagged 60 of 990 salinity observations (6.06%), leaving 930 observations for the primary analysis. The QC-cleaned pooled salinity calibration achieved R^2^ = 0.99771 and RMSE = 3.02 cm, with EC-specific RMSE values of 2.08–2.45 cm. The repeated-measures analysis identified significant main effects of depth and EC but no statistically significant Depth × EC interaction. The maximum mean relative ADC deviation from the EC = 0.5 dS m^−1^ reference was 1.82% at EC = 4.0 dS m^−1^ and 200 cm. Field validation showed strong linear association with the reference instruments and small mean differences; for groundwater, Bland–Altman analysis additionally quantified the limits of agreement. Communication reached the >90% packet-delivery threshold at 8 km in open field with SF12 and at 5 km in dense sugarcane. The routine node energy requirement was 16.60 mWh day^−1^ with BLE disabled during scheduled sensing; the modeled every-wake-up BLE condition was 46.69 mWh day^−1^.

Overall, the reconciled results define a reproducible performance envelope for the integrated sensor network while making the laboratory-versus-field geometry, repeated-measurement structure, communication timing, and BLE operating mode explicit. Future work should expand the number of independently fabricated sensor units and solution-batch replicates, repeat the groundwater calibration with additional sensor units over the full 0–200 cm range, document packet timestamps and independent communication replicates for link-performance trials, and evaluate the BLE-disabled routine energy mode over longer field deployments. Controlled comparisons with conventional FR4-based capacitive probes and other groundwater-level sensing technologies are also needed to quantify the specific performance contributions of the proposed materials and sensor geometries.

## Figures and Tables

**Table 1:** Two-factor within-sensor repeated-measures ANOVA of groundwater-sensor ADC response to water depth and electrical conductivity.

Effect	F-Value	df (Numerator, Denominator)	*p*-Value	Partial η^2^
Water depth	81,338.52	10, 110	<0.001	0.9999
Electrical conductivity	347.95	2, 22	<0.001	0.9694
Depth × EC	0.065	20, 220	1.000	0.0059

**Table 2:** Holm-adjusted paired comparisons among EC levels using sensor-level marginal ADC means averaged across depth.

Comparison	Mean Difference (ADC)	95% CI (ADC)	t(11)	Holm-Adjusted *p*	Cohen’s dz
2.0–0.5 dS m^−1^	15.55	12.61 to 18.48	11.66	<0.001	3.37
4.0–0.5 dS m^−1^	36.61	33.77 to 39.46	28.30	<0.001	8.17
4.0–2.0 dS m^−1^	21.07	17.68 to 24.46	13.68	<0.001	3.95

**Table 3:** Sensor-level descriptive statistics of ADC response after QC and averaging repeated readings.

Depth (cm)	EC = 0.5 dS m^−1^	EC = 2.0 dS m^−1^	EC = 4.0 dS m^−1^	CV Range (%)
	**Mean ± SD**			
0	3683.29 ± 5.62	3698.97 ± 2.31	3720.36 ± 2.42	0.06–0.15
20	3466.03 ± 13.10	3481.78 ± 10.59	3500.57 ± 27.22	0.30–0.78
40	3266.57 ± 29.36	3282.78 ± 3.59	3303.92 ± 11.07	0.11–0.90
60	3064.82 ± 9.38	3079.43 ± 21.59	3098.42 ± 19.46	0.31–0.70
80	2885.71 ± 6.78	2899.53 ± 10.23	2922.94 ± 23.43	0.23–0.80
100	2714.71 ± 7.67	2729.76 ± 13.66	2750.47 ± 8.13	0.28–0.50
120	2564.26 ± 2.96	2581.03 ± 9.30	2601.71 ± 5.32	0.12–0.36
140	2419.96 ± 1.58	2434.90 ± 10.37	2457.51 ± 13.84	0.07–0.56
160	2285.64 ± 3.00	2301.24 ± 2.44	2322.53 ± 3.16	0.11–0.14
180	2164.58 ± 2.31	2180.17 ± 8.18	2202.56 ± 1.70	0.08–0.38
200	2055.74 ± 2.07	2072.72 ± 5.75	2093.07 ± 2.75	0.10–0.28

**Table 4:** Pooled and EC-specific quadratic calibration models for the salinity experiment and comparison of prediction errors.

Model	EC (dS m^−1^)	Calibration Equation	R^2^	RMSE (cm)	Pooled-Model Comparison
Pooled	All levels	2.77 × 10^−5^·ADC^2^ − 0.28·ADC + 659.86	0.9977	3.02	—
EC-specific	0.5	2.79 × 10^−5^·ADC^2^ − 0.28·ADC + 656.96	0.9989	2.08	RMSE 3.03; bias + 2.15; *p* < 0.001
EC-specific	2.0	2.77 × 10^−5^·ADC^2^ − 0.28·ADC + 659.96	0.9986	2.40	RMSE 2.41; bias + 0.21; *p* = 0.170
EC-specific	4.0	2.80 × 10^−5^·ADC^2^ − 0.28·ADC + 667.95	0.9985	2.45	RMSE 3.57; bias − 2.51; *p* < 0.001

Note: Pooled-model error metrics are evaluated against measured depth within each EC subset. Bias is defined as predicted depth minus measured depth. Paired *p*-values are Wilcoxon signed-rank tests comparing absolute errors from the pooled and EC-specific models on the same QC-cleaned observations.

**Table 5:** Depth-interval RMSE of the pooled salinity calibration model after QC.

Depth Interval (cm)	EC = 0.5 dS m^−1^ (cm)	EC = 2.0 dS m^−1^ (cm)	EC = 4.0 dS m^−1^ (cm)	All EC (cm)
0–50	2.53 ± 0.67	1.67 ± 0.13	2.81 ± 0.75	2.43 ± 0.37
51–100	2.19 ± 0.49	1.81 ± 0.63	2.91 ± 1.33	2.45 ± 0.51
101–150	3.46 ± 0.22	1.74 ± 0.58	2.29 ± 0.56	2.62 ± 0.28
151–200	2.80 ± 0.26	1.73 ± 0.44	3.84 ± 0.23	2.94 ± 0.12

**Table 6:** Linear calibration parameters for the capacitive soil moisture sensor at three packing-depth treatments in loam soil.

Depth (cm)	Slope (a)	Intercept (b)	R^2^	RMSE (% *v*/*v*)	95% CI for Slope	95% CI for Intercept
15	0.011468	−3.103	0.9749	1.66	[0.010585, 0.012351]	[−5.037, −1.168]
30	0.009569	−1.118	0.9881	1.08	[0.009065, 0.010072]	[−2.348, 0.112]
45	0.007889	3.108	0.9878	1.09	[0.007469, 0.008310]	[2.057, 4.160]

Notes: Slope units: % *v*/*v* per ADC unit; intercept units: % *v*/*v*. RMSE = root mean square error. Confidence intervals are OLS model intervals.

**Table 7:** ANCOVA results for testing depth-dependence of soil moisture sensor calibration parameters.

Source of Variation	SS	DOF	MS	F-Value	*p*-Value
ADC	6164.228	1	6164.228	3281.20	<0.001
Depth	32.791	2	16.395	8.73	<0.001
Depth × ADC	146.388	2	73.194	38.96	<0.001
Error (Residual)	107.083	57	1.879	—	—
Total	6450.490	62	—	—	—

Notes: DC = analog-to-digital converter output (0–4095). SS = sum of squares; DOF = degrees of freedom; MS = mean square.

**Table 8:** Statistical comparison of capacitive soil moisture sensors versus TDR350 reference measurements (*n* = 90 per depth).

Depth (cm)	Capacitive Mean (% *v*/*v*)	TDR Mean (% *v*/*v*)	Mean Difference	SD of Difference	Paired *t*-Test *p*-Value	Cohen’s d
15	18.44 ± 7.84	17.75 ± 7.78	+0.69	2.36	0.0067 (Holm 0.0200)	0.29
30	29.32 ± 7.69	29.00 ± 7.61	+0.31	1.89	0.1215 (Holm 0.1215)	0.16
45	21.14 ± 7.88	20.67 ± 7.84	+0.47	2.10	0.0363 (Holm 0.0726)	0.22

**Table 9:** Linear regression parameters for capacitive soil moisture sensors versus TDR350 reference.

Depth (cm)	Regression Equation	R^2^	RMSE (% *v*/*v*)	Slope (95% CI)	Intercept (95% CI)
15	θ_cap_ = 0.961 × θ_TDR_ + 1.4	0.911	2.33	0.961 [0.897, 1.025]	1.4 [0.2, 2.6]
30	θ_cap_ = 0.980 × θ_TDR_ + 0.9	0.940	1.88	0.980 [0.927, 1.032]	0.9 [−0.7, 2.5]
45	θ_cap_ = 0.969 × θ_TDR_ + 1.1	0.930	2.08	0.969 [0.913, 1.026]	1.1 [−0.1, 2.4]

θ_cap_ and θ_TDR_ represent soil moisture measured by a capacitive sensor and TDR, respectively.

**Table 10:** Statistical comparison of the capacitive groundwater sensor versus dip-meter reference measurements (*n* = 90 daily paired observations).

Metric	Value
Capacitive Sensor Mean (cm)	89.14 ± 23.42
Dip-Meter Mean (cm)	88.74 ± 23.94
Mean Difference (Bias)	+0.397 cm
Standard Deviation of Difference	2.002 cm
Paired *t*-test	1.88
Degrees of Freedom	89
*p*-value (two-tailed)	0.063
Cohen’s d	0.20
95% CI for Mean Difference	[−0.023, +0.816] cm

**Table 11:** Regression and agreement analysis for the capacitive groundwater sensor versus dip-meter reference.

Analysis Type	Parameter	Value
Linear Regression	Equation	h_cap_ = 0.9747 × h_dip_ + 2.6402
	R^2^	0.9934
	RMSE (cm)	1.90
	Slope (95% CI)	0.9747 [0.9578, 0.9916]
	Intercept (95% CI)	2.64 [1.09, 4.19]
Bland–Altman	Mean Bias (cm)	+0.397
	Standard Deviation of Differences (cm)	2.002
	95% Limits of Agreement (cm)	−3.53 to +4.32
	Proportion within Limits (%)	93.3

**Table 12:** LoRaWAN communication performance by distance, spreading factor, and vegetation condition (*n* = 100 uplinks per condition).

Distance	Condition	SF	PDR (%)	Mean RSSI (dBm)	Mean SNR (dB)	Airtime (ms)	Link Status
1	Open field	7	100 ± 0.0	−98.2 ± 2.1	14.3 ± 1.1	41	Reliable
		9	100 ± 0.0	−104.6 ± 2.4	11.2 ± 1.3	164	Reliable
		12	100 ± 0.0	−111.3 ± 2.7	8.1 ± 1.4	655	Reliable
5	Open field	7	82.4 ± 4.9	−119.8 ± 3.8	1.2 ± 1.9	41	Degraded
		9	96.3 ± 2.1	−124.2 ± 3.4	−2.8 ± 1.7	164	Reliable
		12	95.8 ± 2.3	−125.6 ± 3.6	−4.6 ± 1.7	655	Reliable
8	Open field	7	38.1 ± 9.2	−131.7 ± 4.5	−5.3 ± 2.4	41	Unreliable
		9	71.6 ± 7.4	−133.9 ± 4.1	−7.1 ± 2.2	164	Degraded
		12	90.3 ± 4.1	−132.4 ± 3.8	−6.2 ± 1.9	655	Threshold
1	Sugarcane	7	98.7 ± 1.1	−100.4 ± 2.6	12.8 ± 1.4	41	Reliable
		9	99.2 ± 0.8	−106.1 ± 2.9	9.7 ± 1.5	164	Reliable
		12	99.1 ± 0.9	−112.8 ± 3.1	6.9 ± 1.6	655	Reliable
5	Sugarcane	7	74.2 ± 6.3	−122.6 ± 4.3	−0.4 ± 2.1	41	Degraded
		9	89.4 ± 3.8	−126.8 ± 3.9	−3.9 ± 1.9	164	Degraded
		12	92.1 ± 2.7	−127.3 ± 4.2	−5.1 ± 2.0	655	Reliable
8	Sugarcane	7	29.3 ± 8.7	−134.2 ± 5.1	−7.8 ± 2.7	41	Unreliable
		9	63.8 ± 8.9	−136.4 ± 4.7	−9.4 ± 2.5	164	Degraded
		12	84.6 ± 5.3	−134.9 ± 4.9	−7.8 ± 2.3	655	Degraded

Notes: PDR = packet delivery ratio; RSSI = received signal strength indicator; SNR = signal-to-noise ratio. Values represent mean ± standard deviation.

**Table 13:** Power consumption by operational state for ESP32-based child node (*n* = 10 measurements per state).

Operational State	Current Draw (mA)	Duration per 20 min Cycle	Energy per Cycle (mJ)
Deep sleep (peripherals off)	0.028 ± 0.003	19 min 58 s (1198 s)	110.7
Sensor sampling (100 ms)	42.3 ± 2.1	0.1 s	14
LoRa transmission (SF12, 11-byte)	118.7 ± 4.3	1.8 s	705.1
BLE advertising scan (30 s, optional)	15.2 ± 1.1	30 s	1504.8
Total per cycle (without BLE)	—	—	829.8
Total per cycle (with BLE)	—	—	2334.6

Note: Energy is calculated as Current (mA) × 3.3 V × Duration (s). For routine sensing with BLE disabled, the calculated energy is 829.8 mJ per 20 min cycle, corresponding to 16.60 mWh day^−1^ at 72 cycles day^−1^. If the 30 s BLE scan is executed at every wake-up, the modeled total becomes 2334.6 mJ per cycle and 46.69 mWh day^−1^.

## Data Availability

The original contributions presented in this study are included in the article. Further inquiries can be directed to the corresponding authors.

## Appendix A

**Table A1:** Bill of Materials (BOM) and Cost Breakdown.

Component	Specification	Unit Cost (USD)
Microcontroller	ESP32-WROOM-32D Development Board	$8.50
LoRa Transceiver	SX1276 Module (868 MHz)	$6.00
Soil Moisture Probe	PMMA substrate, PTFE-insulated copper wire, epoxy	$4.50
Groundwater Probe	19 mm PVC pipe, bare stainless steel wire, sand filter	$3.00
Power Storage	3.7 V, 10,000 mAh Li-Po Battery	$15.00
Energy Harvesting	2 W Monocrystalline Solar Panel	$12.00
Enclosure	IP67-rated ABS box with cable glands	$10.00
Power Management	TP4056 charger, TPS22860 load switch, voltage dividers	$8.00
Miscellaneous	3 dBi Antenna, 32.768 kHz RTC, micro-SD, solder, hardware	$7.00
Total Estimated Cost per Node		$74.00

**Table A2:** LoRaWAN Radio Configuration Settings.

Parameter	Setting	Justification
Frequency Band	868.1 MHz	Standard EU ISM band for long-range rural coverage.
Modem	SX1276 (Semtech)	Supports 137–1020 MHz.
Bandwidth (BW)	125 kHz	Optimal balance between receiver sensitivity and airtime.
Spreading Factor (SF)	SF7 to SF12 (Adaptive)	SF7 for <1 km; SF12 for 5–8 km or dense vegetation.
Coding Rate (CR)	4/5	Standard forward error correction for agricultural noise.
Transmit Power	14 dBm	Complies with EIRP limits while maximizing range.
Preamble Length	8 symbols	Standard ChirpStack compatibility.
Sync Word	0x12	Designates a private LoRaWAN network.
